# Revision of the East Mediterranean *Orthomus* (Coleoptera, Carabidae, Pterostichini), with description of *Parorthomus* gen. n. *socotranus* sp. n. from Socotra Island and key to the Old World genera of subtribe Euchroina

**DOI:** 10.3897/zookeys.427.7618

**Published:** 2014-07-21

**Authors:** Borislav Guéorguiev, David W. Wrase, Jan Farkač

**Affiliations:** 1 National Museum of Natural History, 1 Blvd. Tzar Osvoboditel, 1000 Sofia, Bulgaria; 2 Dunckerstr. 78, D-10437 Berlin, Germany; 3 Czech University of Life Sciences Prague, Faculty of Forestry and Wood Sciences, Kamýcká 129, CZ-165 21, Praha 6 – Suchdol, Czech Republic

**Keywords:** Coleoptera, Carabidae, Pterostichini, *Orthomus*, *Parorthomus*, taxonomy, new genus, new species, new synonyms, lectotype designation, key, checklist, East Mediterranean, Spain, Republic of Yemen

## Abstract

The East Mediterranean species of *Orthomus* Chaudoir, 1838 are revised. The type series of *Feronia longula* Reiche & Saulcy, 1855, *F. berytensis* Reiche & Saulcy, 1855, *F. proelonga* Reiche & Saulcy, 1855, *Orthomus longior* Chaudoir, 1873, *O. sidonicus* Chaudoir, 1873, and *O. berytensis akbensis* Mateu, 1955 were studied and lectotypes for the first four are designated. Also, the following nomenclatural acts are proposed: *Feronia proelonga* Reiche & Saulcy, 1855, **syn. n.** of *Orthomus berytensis* (Reiche & Saulcy, 1855); *Feronia elongata* Chaudoir, 1859, **syn. n.** of *Orthomus berytensis* (Reiche & Saulcy, 1855); *Orthomus sidonicus* Chaudoir, 1873, **syn. n.** of *Orthomus longior* Chaudoir, 1873; *Orthomus velocissimus andalusiacus* Mateu, 1957, **syn. n.** of *Orthomus velocissimus akbensis* Mateu, 1955, new assignment for *Orthomus berytensis akbensis* Mateu, 1955. As a result, three species of the genus inhabit the East Mediterranean biogeographical region: *O. berytensis*, *O. longior*, and *O. longulus*. A key to these three species is given. *O. longior* is recorded for Turkey and Syria for the first time. In addition, a new synonymy of two West Mediterranean taxa is proposed: *O. szekessyi* (Jedlička, 1956), **syn. n.** of *O. balearicus* (Piochard de la Brûlerie, 1868), and a new genus and a species are described: *Parorthomus* **gen. n.** *socotranus* **sp. n.** (type locality: Republic of Yemen, Socotra Archipelago, Socotra Island, Fimihin env., 530 m.a.s.l.). Illustrations of the species dealt with here are provided including external characters, habitus, mentum and submentum, and genitalia are provided.

Nine genera of the “African Series” of subtribe Euchroina Chaudoir, 1874 are keyed for the first time. Checklists of the species of *Orthomus* and of the Old World euchroine genera are given.

## Introduction

*Orthomus* Chaudoir, 1838 is a Palaearctic genus of pterostichine carabid beetles with Circum-Mediterranean distribution, which includes 23 species (Bousquet 2003, Lorenz 2005, Wrase and Jeanne 2005, Pupier and Coulon 2013). The greatest diversity of species is concentrated in the Western Mediterranean, mostly in the Iberian Peninsula and North-West Africa. Only a few species live in the Eastern Mediterranean. The group is often ranked as nominotypical subgenus of *Orthomus* s.l., together with the Macaronesian *Eutrichopus* Tschitschérine, 1897 (three species), *Gietopus* Machado, 1992 (one species), *Nesorthomus* Bedel, 1899 (eight species), *Wolltinerfia* Machado, 1985 (two species), and the North-West African *Trichopedius* Bedel, 1899 (one species) (Bousquet ibid., Lorenz ibid., Serrano et al. 2009, Serrano et al. 2012), but some authors accepted a generic status (Mateu 1954, Machado 1992, Ortuño 1996, Wrase and Jeanne 2005, Donabauer 2008).

A recent work on mitochondrial DNA variations among species of *Eutrichopus*, *Wolltinerfia* and *Orthomus*, found relatively high genetic divergence between them and suggested dealing with these as distinct genera (Moya et al. 2004: 3163). Based on established phyletic distance, the last taxon is treated here as an independent genus.

All the afore-enumerated taxa together with the Afrotropical genera *Abacillius* Straneo, 1949 and *Abacillodes* Straneo, 1988 were referred as to the “African Series” of subtribe Euchroina Chaudoir, 1874 (Will 2006). The species included in Euchroina share several derived characters that is exceedingly short or no coronal suture (i), ventrally extended membranous band on the maxillary stipes (ii) in the larvae (Bousquet and Liebherr 1994), well-impressed frontal furrows of head, not or hardly attaining the level of anterior supra-orbital puncture (iii), hind trochanters without setae (iv), and “gooseneck” shaped bursa copulatrix (v) in the imago (Will 2002, 2006, Frania and Ball 2007), which suggest monophyletic origin of the subtribe. Besides, most Old World euchroines have relatively large and prominent eyes compared to the size of the head, submentum without lateral setae, distinct parascutellar stria, sternites V-VII with transverse sulci (complete or not), segment 5 of tarsomeres setose beneath and median lobe of aedeagus with dorsal ostium.

The genus *Cedrorum* Borges & Serrano, 1993 from the Azores does not belong to this series as its only species, *Cedrorum azoricus* Borges & Serrano, 1993, exhibits a set of features atypical of the euchroines, such as: superficial frontal furrows, submentum with lateral setae, left paramere with a slight transverse aphophysis, falcate right paramere, no transverse sulci on sternites V-VII (contrary to the statement of Borges and Serrano 1993: 320) and segment 5 of tarsomeres glabrous ventrally.

The East Mediterranean species of *Orthomus* were the object of taxonomic reviews quite a while ago (Mateu 1955). Since then, however, new data and new material have accumulated. Investigations of material in several European museum and private collections demonstrated that the identifications of the species from the area in question were often incorrect, our investigations proved the occurrence of only three species there. For example, a review of material from Greece, formerly published or identified as *Orthomus barbarus* and *Orthomus berytensis*, revealed that only the latter species inhabits this country (Arndt et al. 2011: 58). Hence, the necessity to improve our taxonomic knowledge of the East Mediterranean representatives of the genus was the main reason to start this work. Besides, a few years ago one of us (DWW) received a series of *Orthomus*-like specimens from Socotra Island. Subsequent examination has proven that it belongs to a species and a genus new to science.

## Material and methods

More than 260 specimens of twenty-four species (and one new to science from Yemen) of Euchroina from the Mediterranean and Afrotropical region were examined: *Abacillius basilewskyi* Straneo, 1962, *Abacillodes jocquei* Straneo, 1988, *Abacillodes malawianus* Straneo, 1988, *Orthomus abacoides* (Lucas, 1846), *Orthomus aquila* (Coquerel, 1859), *Orthomus aubryi* Jeanne, 1974, *Orthomus balearicus* (Piochard de la Brûlerie, 1868), *Orthomus barbarus barbarus* (Dejean, 1828), *Orthomus barbarus formenterrae* (Breit, 1933), *Orthomus barbarus penibeticus* Mateu & Colas, 1954, *Orthomus berytensis* (Reiche & Saulcy, 1855), *Orthomus dimorphus dimorphus* Antoine, 1933, *Orthomus dimorphus antoinei* Mateu, 1955, *Orthomus discors* (Wollaston, 1864), *Orthomus hispanicus* (Dejean, 1828), *Orthomus lacouri lacouri* (Antoine, 1941), *Orthomus lacouri kocheri* Mateu, 1955, *Orthomus leprieuri* Pic, 1894, *Orthomus longior* Chaudoir, 1873, *Orthomus longulus* (Reiche & Saulcy, 1855), *Orthomus maroccanus* Chaudoir, 1873, *Orthomus perezii* (Martinez & Saez, 1873), *Orthomus planidorsis* (Fairmaire, 1872), *Orthomus rubicundus* (Coquerel, 1859), *Orthomus starkei* Wrase & Jeanne, 2005, *Orthomus tazekensis tazekensis* (Antoine, 1941), *Orthomus tazekensis rifensis* Wrase & Jeanne, 2005, *Orthomus velocissimus velocissimus* (Waltl, 1835), *Orthomus velocissimus akbensis* Mateu, 1955, *Orthomus velocissimus pardoi* Mateu, 1957. For comparison we examined also several specimens of *Cedrorum azoricus azoricus* Borges & Serrano, 1993 and of *Cedrorum azoricus caveirensis* Borges & Serrano, 1993, a species not belonging to the euchroines.

The lectotypes are designated and validated in order to stabilize the nomenclature in the genus according to Article 74.7.3 of the Code (ICZN 1999). Without this action, our concept of some “well-known” species would be uncertain, because in many instances, we dealt with type-series consisting of two or more species, and the series are divided between two museums.

Male specimens were boiled in water and their genitalia were extracted, put in 10% KOH solution, then washed and neutralized and then stored in glycerine. The figures were made with Zeiss transmitted-light microscope. After that, the aedeagus and parameres were embedded in Euparal either on the same card with specimen from which they were extracted or on a separate transparent label beneath the specimen from whom they were removed. The measurements and other drawings were made with the aid of an Olympus SZX10 stereobinocular. The photos of habitus and pronotum were made with a Zeiss Stemi 2000 microscope equipped with an AxioCam ERc 5s camera.

Measurements: body length from the apex of the longer mandible to the apex of the longer elytron (BL); maximum linear distance across the head, including the eyes (HW); maximum width of pronotum (PW); length of pronotum, measured from the apical margin to the basal margin along the midline (PL); width of the pronotal base, between the tips of the hind angles (PbW); length of elytra, from a line connecting the apices of the humeral angles to the apex of the longer elytron (EL); maximum width of elytra (EW). The surface of the paramere close-fitting to the distal part of the median lobe of the aedeagus is denoted as internal face, on the contrary the second surface is the external face.

The material examined is housed in the collections listed below:

BMNH The Natural History Museum, London, United Kingdom (Max Barkley)

CRHG Christoph Reuter collection, Hamburg, Germany

DWBG David W. Wrase collection, Berlin, Germany

HLMD Hessisches Landesmuseum Darmstadt, Darmstadt, Germany (Sabine Wamser)

IRSNB Institut Royal des Sciences Naturelles de Belgique, Brussels, Belgium (Alain Drumont)

JFPC Jan Farkač collection, Prague, Czech Republic

JSAG Joachim Schmidt collection, Admannshagen, Germany

MBAP Museo del Dipartimento di Biologia Animale dell’Università, Pavia, Italy (Edoardo Razzetti, Stefano Maretti)

MHNG Muséum d’histoire naturelle, Genève, Switzerland (Julio Cuccodoro)

MIZ Museum and Institute of Zoology, Polish Academy of Sciences, Warszawa, Poland (Dominika Mierzwa)

MNH Entomologie, Muséum National d'Histoire Naturelle, Paris, France (Thierry Deuve, Azadeh Taghavian)

MPHG Manfred Persohn collection, Herxheimweyer, Germany

MRAC Musee Royal de l’Afrique Centrale, Tervuren, Belgium (Marc De Meyer)

NMNHS National Museum of Natural History, Sofia, Bulgaria (Borislav Guéorguiev)

NMPC Národní museum v Prague, Prague, Czech Republic (Jiří Hájek)

NMW Naturhistorisches Museum Wien, Vienna, Austria (Harald Schillhammer)

PSHG Peer H. Schnitter collection, Halle, Germany

RFBN Ron Felix collection, Berkel-Enschot, the Netherlands

TAU National Collection of Insects, Department of Zoology, Tel Aviv University, Israel (Laibale Friedman)

TFPG Thomas Forcke collection, Pforzheim, Germany

VSKC Vladimir Skoupý collection, Kamenné Žehrovice, Czech Republic

ZMAN Zoölogisch Museum Amsterdam, the Netherlands (Ben Brugge)

## Taxonomy

### Key to the Old World genera of Euchroina

**Table:** 

1	Tarsomeres of all legs glabrous dorsally. Elytra with parascutellar striae well-engraved (long in *Orthomus*, *Abacillodes*, *Parorthomus* gen. n., short in *Abacillius*)	2
–	Tarsomeres of all legs pubescent dorsally. Elytra with parascutellar striae vestigial or lacking	6
2	Elytra with discal setiferous punctures in interval 3 / stria 3. Mentum tooth bifid	3
–	Elytra without discal setiferous punctures. Mentum tooth hardly excavate (*Abacillodes aculeatus*) or round (*Abacillius basilewskyi*) at tip	*Abacillius* Straneo, 1949
3	Elytra with two setiferous punctures in interval 3 / stria 3, with last puncture in medial third of elytron	4
–	Elytra with three to four setiferous punctures in interval 3 / stria 3, with last puncture in posterior third of elytron	*Parorthomus* gen. n.
4	Pronotum subquadrate (sides straight or slightly narrowed towards hind angles), with anterior angles slightly to moderately prominent forward. Abdominal sternites V-VII with transverse sulci complete and well-impressed	5
–	Pronotum subtrapezoid (sides broadened towards hind angles), with fore angles fairly prominent forward. Abdominal sternites V-VII with transverse sulci distinct only laterally (*Nesorthomus annae* Donabauer, 2008, *Nesorthomus bedelianus* Lutshnik, 1915 and *Nesorthomus dilaticollis* Wollaston, 1854) or lacking (remaining species)	*Nesorthomus* Bedel, 1899
5	Species with intercoxal process of prothorax bordered and distribution in the Mediterranean biogeographical region	*Orthomus* Chaudoir, 1838
–	Species with intercoxal process of prothorax slightly bordered (*Abacillodes jocquei*) or unbordered (*Abacillodes malawianus*) and distribution in the Afrotropical biogeographic region (Malawi)	*Abacillodes* Straneo, 1988
6	Smaller species (less than 6 mm), with continental distribution in northwest Africa	*Trichopedius* Bedel, 1899
–	Larger species (more than 6 mm), with insular distribution in the Macaronesian biogeographical region	7
7	Head with distinct eyes and paraorbital sulci not extended behind posterior margin of eye. Elytra truncate apically	*Eutrichopus* Tschitschérine, 1897
–	Head with very small or no eyes and paraorbital sulci grooved, extended behind posterior margin of eye toward neck. Elytra rounded apically	8
8	Sides of pronotum straight towards hind angles. Elytra not narrowed basally	*Gietopus* Machado, 1992
–	Sides of pronotum distinctly concave towards hind angles. Elytra narrowed basally	*Wolltinerfia* Machado, 1985

### Checklist of the Old World genera of Euchroina

***Abacillius*** Straneo, 1949 – Republic of South Africa

***Abacillodes*** Straneo, 1988 – Malawi

***Eutrichopus*** Tschitschérine, 1897 – Canary Islands

***Gietopus*** Machado, 1992 – Canary Islands

***Nesorthomus*** Bedel, 1899 – Madeira

***Orthomus*** Chaudoir, 1838 – Canary Islands, Mediterranean

***Parorthomus*** gen. n. – Socotra

***Trichopedius*** Bedel, 1899 – Algeria

***Wolltinerfia*** Machado, 1985 – Canary Islands

### I. Revision of the East Mediterranean *Orthomus* species

#### Orthomus berytensis

Taxon classificationAnimaliaColeopteraCarabidae

(Reiche & Saulcy, 1855)

**Figures 1–3.**
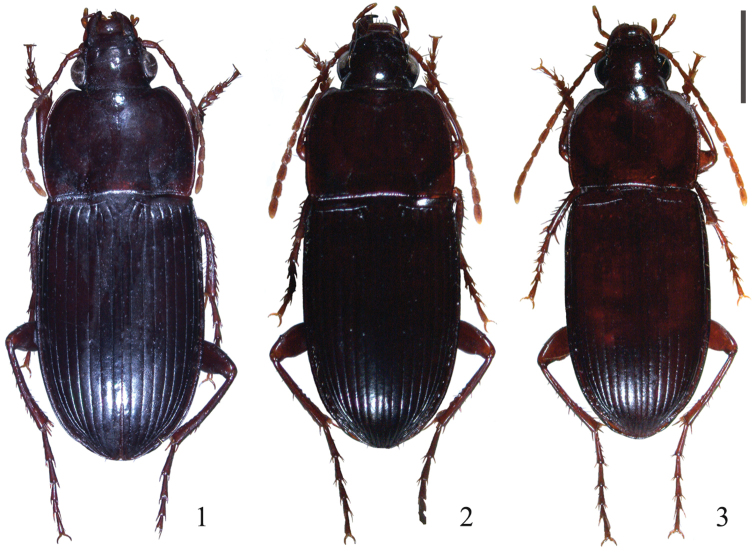
Habitus. **1** *Orthomus berytensis* (Reiche & Saulcy, 1855), male, “Tel-Aviv” **2** *Orthomus longior* Chaudoir, 1873, male, “Upper Galilee, Ya’ar Bar’am” **3** *Orthomus longulus* (Reiche & Saulcy, 1855), male, “Upper Galilee, Ha Khula Valley, Ma’agar Einan lake”. Scale bar = 2 mm.

**Figures 4–7.**
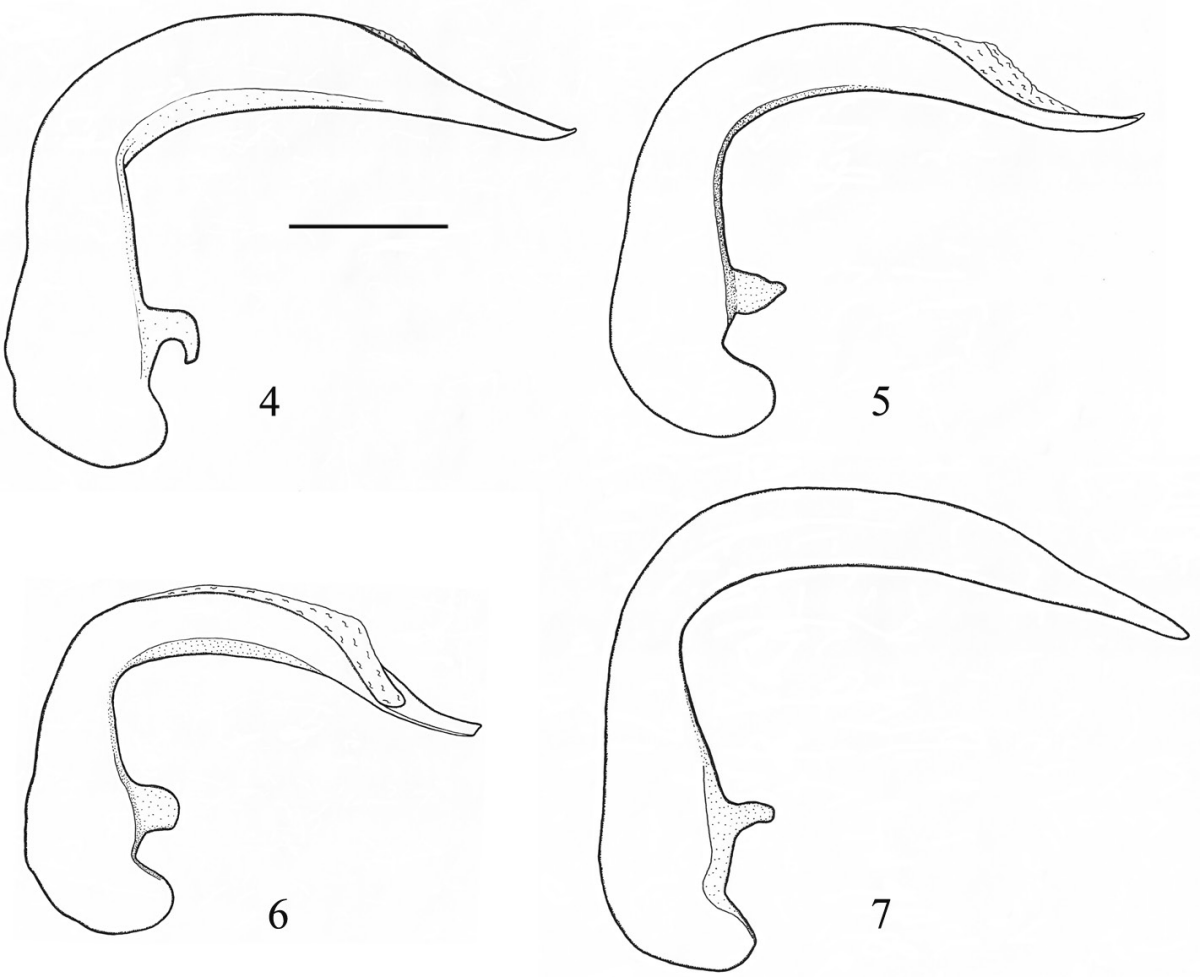
Median lobe of aedeagus, lateral view. **4** *Orthomus berytensis* (Reiche & Saulcy, 1855), lectotype of *Feronia berytensis* **5** *Orthomus longior* Chaudoir, 1873, paralectotype of *Feronia longula* Reiche & Saulcy **6** *Orthomus longulus* (Reiche & Saulcy, 1855), male, “Megiddo” **7** *Orthomus velocissimus akbensis* Mateu, 1955, holotype of *Orthomus barbarus akbensis*. Scale bar = 0.5 mm.

**Figures 8–11.**
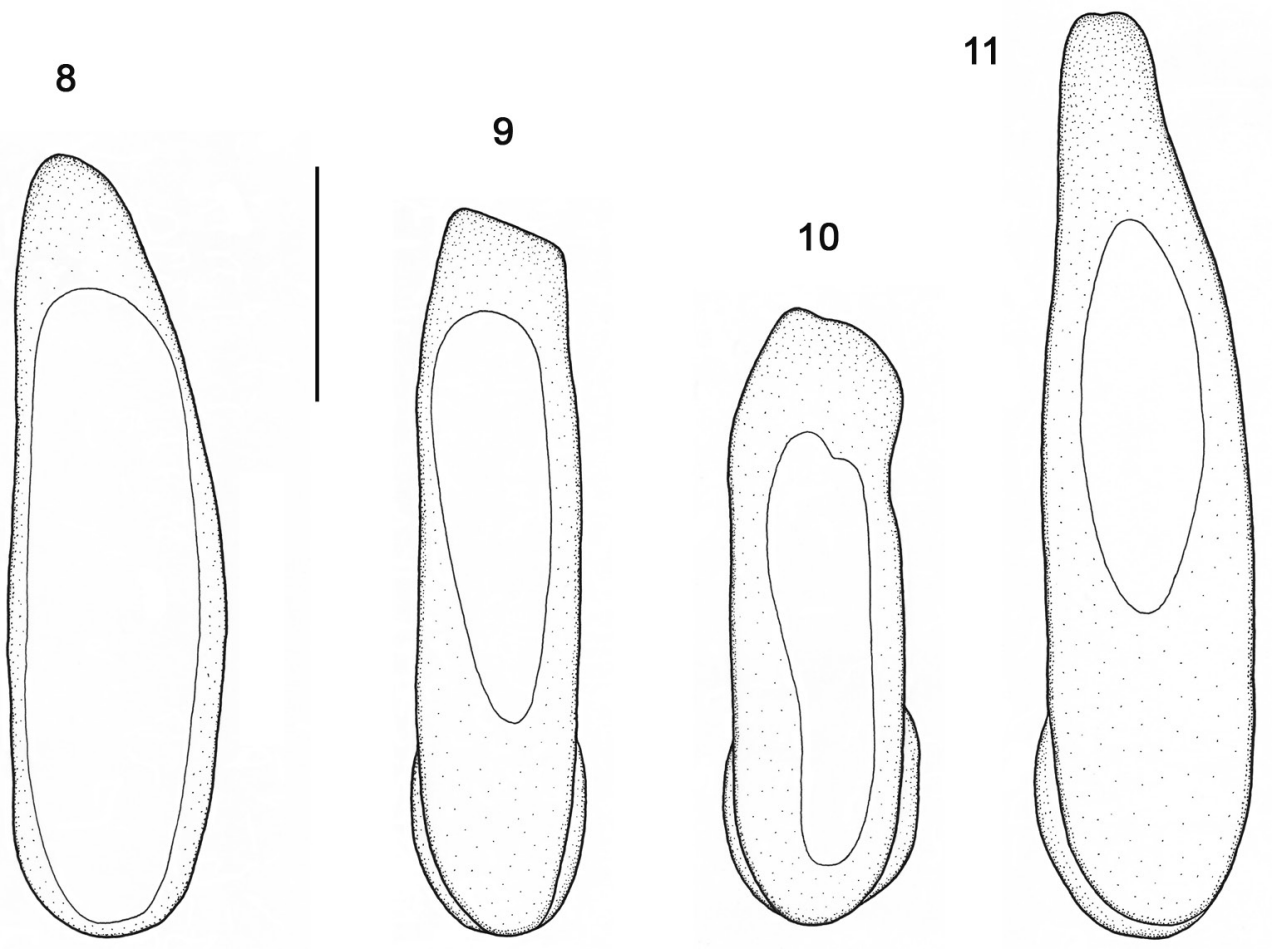
Median lobe of aedeagus, dorsal view. **8** *Orthomus berytensis* (Reiche & Saulcy, 1855): lectotype of *Feronia berytensis* **9** *Orthomus longior* Chaudoir, 1873, paralectotype of *Feronia longula* Reiche & Saulcy **10** *Orthomus longulus* (Reiche & Saulcy, 1855), male, “Megiddo” **11** *Orthomus velocissimus akbensis* Mateu, 1955, holotype of *Orthomus barbarus akbensis*. Scale bar = 0.5 mm.

**Figures 12–15.**
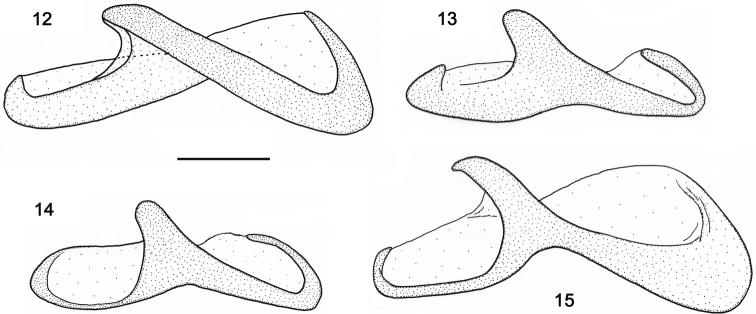
Right paramere, internal face. **12** *Orthomus berytensis* (Reiche & Saulcy, 1855): lectotype of *Feronia berytensis* **13** *Orthomus longior* Chaudoir, 1873, paralectotype of *Feronia longula* Reiche & Saulcy **14** *Orthomus longulus* (Reiche & Saulcy, 1855), male, “Megiddo” **15** *Orthomus velocissimus akbensis* Mateu, 1955, holotype of *Orthomus barbarus akbensis*. Scale bar = 0.2 mm.

**Figures 16–17.**
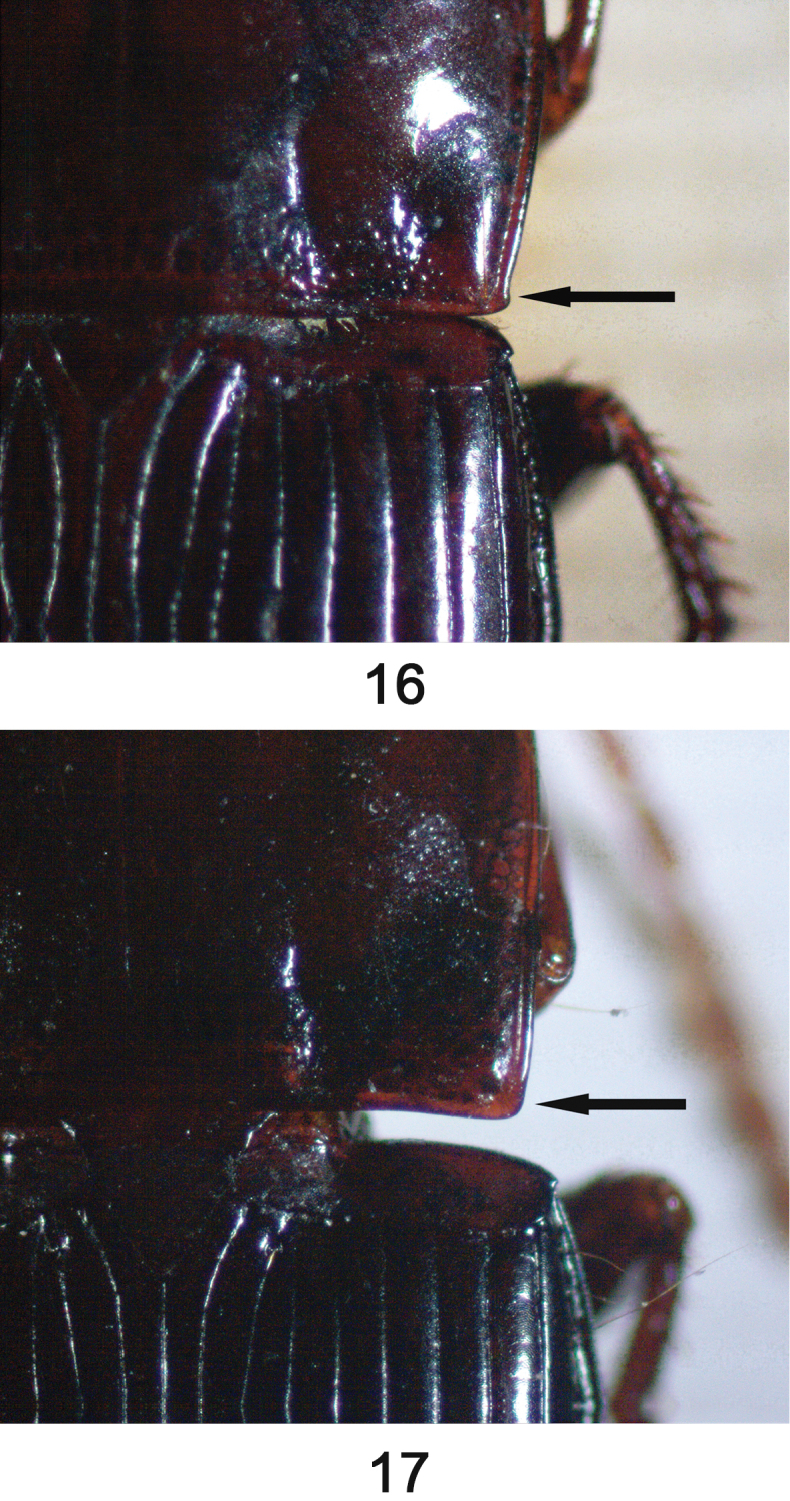
Pronotum posterior right angle. **16** *Orthomus berytensis* (Reiche & Saulcy, 1855), male, “Tel-Aviv” **17** *Orthomus longior* Chaudoir, 1873, male, “Upper Galilee, Ya’ar Bar’am”.

Figs 1 , 4 , 8 , 12 , 16

Feronia (Argutor) berytensis Reiche & Saulcy, 1855: 618 (type locality: “De Beyrouth”)
Feronia (Argutor) longula Reiche & Saulcy, 1855: 616, part
Feronia (Argutor) proelonga Reiche & Saulcy, 1855: 619 (type locality: “Des bords du Jourdain”), syn. n.
Feronia elongata Chaudoir, 1859: 116 (type locality: “Moräa, Beyruth und Alexandrien.”), syn. n.
Feronia varinii ? Gautier des Cottes, 1866: 178 (type locality: “Sardinia”)
Feronia atlantica Fairmaire, 1875: 543 (type locality: “Mogador” [Essaouira, Morocco])
Othomus [sic] *longior* Chaudoir, 1873: 105, part
Feronia (Orthomus) barbara : Piochard de la Brûlerie, 1876: 416
Orthomus barbarus berytensis : Mateu, 1955: 56, 76
Orthomus muluyensis Antoine, 1957: 205 (type locality: Guercif)
Pterostichus (Orthomus) haligena Wollaston, 1860: 87 (type locality: “Great Salvage”)

##### Note.

The taxon *haligena*, described from the Salvage Islands belongs to *Orthomus berytensis* (see Machado 1992: 260, Lorenz 1998: 249, 2005: 265) and not to *Orthomus barbarus* where it was quoted by Bousquet (2003: 477), as a subspecies, erroneously with the distribution data Madeira. The examination of 13 specimens (Ilhas Selvagens, Selvagem Grande, SE. sublittoral zone, 65-90 m (under stones), XI/XII 2006, D. Putzer leg., DWBG) in comparison with specimens from the Canaries and from Morocco revealed some differences, namely a smaller body size and a strong elytral reticulation in males, almost as strong as in females. These differences could suggest that we deal with a distinct taxon but further investigations are needed. The taxonomic status of the names *atlantica* and *muluyensis*, both partly considered in the literature as a “forma” or a subspecies of *berytensis* or as a synonym to this name must be cleared up by further investigation of a larger sample of material.

##### Type material.

***Feronia berytensis* Reiche & Saulcy, 1855.** Consists of six syntypes, 4 ♂♂, 1 ♀ preserved in MHNG and 1 ♂ in MNHP. The study revealed conspecificity. The specimens in MHNG are placed under a Melly’s taxa label “*longulus* Reiche, var: *berytensis* Reiche. ” [handwritten by Melly with pen]. Further, all specimens bear individually the following two labels “Coll. Reiche” [black print on white label] and “*Orthomus longulus* Reiche var. *berytensis* Reiche Label MHNG 2010” [black print on white label by Cuccodoro]. In addition, the male selected as lectotype with label, subsequently added: “Lectotype *Feronia berytensis* Reiche & Saulcy, 1855 B.Guéorguiev & D.W.Wrase des. 2012” [black print on red label], the remaining five: “Paralectotype *Feronia berytensis* Reiche & Saulcy, 1855 B.Guéorguiev & D.W.Wrase des. 2012” [black print on red label]. The genitalia of the lectotype and of a further male paralectotype from MHNG were examined and glued to cards, pinned beneath the specimens from which they had been removed. The specimen from MNHP is a male, with previously extracted genitalia and glued on a separate card pinned beneath the specimen. This sample bears two old labels equal in size and type: “*berytensis* type Reiche” [handwritten on white label by Mateu], “Syrie” [handwritten on white label by Chaudoir], as well as a new one: “Paralectotype *Feronia berytensis* Reiche & Saulcy, 1855 B.Guéorguiev & D.W.Wrase des. 2012” [black print on red label]. All six specimens with a label, subsequently added: “*Orthomus berytensis* (Reiche & Saulcy) det. B.Guéorguiev” [black print on white label].

We got the information that the part of the Reiche collection housed in MHNG was integrated into the general collection (which was build up essentially from the collection of André Melly) with all specimens with: “Coll. Reiche” but usually there are neither locality, nor identification labels attached to individual specimens, and that identification and locality data figure only on the 'taxa labels' (handwritten by Melly) pinned to the bottom of the drawer, which have thus to be considered as pertaining collectively to the specimens pinned above (Cuccodoro in litt.). The species was described from “Beyruth”, taking into consideration the above mentioned facts concerning labelling of the types we suppose the above mentioned typical specimens as coming from the type locality.

**Type material. *Feronia longula* Reiche & Saulcy, 1855 (specimens belonging to *Orthomus berytensis*).** The type series in MHNG contains two specimens belonging to *Orthomus berytensis*: 1 ♀, “Jaffa, Syrie” [Reiche's handwriting on yellow label], “Coll. Reiche” [black print on white label], “*Orthomus longulus* Reiche Label MHNG 2010” [black print on white label by G. Cuccodoro]; 1 ♂, “Coll. Reiche” [black print on white label], “*Orthomus longulus* Reiche ‘Egypte, Syrie.’ Label MHNG 2010” [black print on white label by G. Cuccodoro], and with labels subsequently added: “Paralectotype *Feronia longula* Reiche & Saulcy, 1855 B.Guéorguiev & D.W.Wrase des. 2012” [black print on red label], “*Orthomus berytensis* (Reiche & Saulcy) det. B.Guéorguiev” [black print on white label].

**Type material. *Feronia proelonga* Reiche & Saulcy, 1855.** Consists of 2 ♂♂, 2 ♀♀ preserved in MHNG and 1 ♂ in MNHP, all conspecific. The specimens in MHNG are placed under a Melly’s taxa label “var: *praelongus* Reiche, Palestine” [handwritten by A. Melly with pen]. One male specimen, chosen for lectotype bears the following labels: “Jourdain” [Reiche's handwriting on yellow label], “Coll. Reiche” [black print on white label], “*Orthomus praelongu* [sic!] Reiche ‘Palestine.’ Label MHNG 2010” [black print on white label by Cuccodoro], “Lectotype *Feronia proelonga* Reiche & Saulcy, 1855 B.Guéorguiev & D.W.Wrase des. 2012” [black print on red label]. One male and two females from MHNG: “Coll. Reiche” [black print on white label], “*Orthomus praelongu* [sic!] Reiche ‘Palestine.’ Label MHNG 2010” [black print on white label by G. Cuccodoro], “Paralectotype *Feronia proelonga* Reiche & Saulcy, 1855 B.Guéorguiev & D.W.Wrase des. 2012” [black print on red label] are accordingly designated. All four specimens with a label, subsequently added: “*Orthomus berytensis* (Reiche & Saulcy) det. B.Guéorguiev” [black print on white label]. The genitalia of the lectotype and the male paralectype from MHNG examined by ourselves (the apices of both median lobes were found damaged!) and glued to cards together with the respective specimens. The specimen from MNHP, designated as paralectotype, was with previously extracted genitalia. Its aedeagus and parameres are glued to a separate card pinned beneath the specimen. Additionally, this paralectotype bears two old labels equal in size and type: “*proelongus* type Reiche” [handwritten on white label by Mateu] and “Jourdain” [handwritten on white label by Chaudoir], as well as a new one: “Paralectotype *Feronia proelonga* Reiche & Saulcy, 1855 B.Guéorguiev & D.W.Wrase des. 2012” [black print on red label]. All specimens with a label, subsequently added: “*Orthomus berytensis* (Reiche & Saulcy) det. B.Guéorguiev” [black print on white label].

The study of the type material of *Feronia proelonga* found that this taxon is to be removed from synonymy with *Orthomus barbarus barbarus* (cfr. Bousquet 2003: 477) and treated as conspecific with *Orthomus berytensis*. Originally, it was separated by the authors in the relatively shorter size of body and pronotum seemingly more transversal, with two basal impressions distinct and strongly punctured. We found such variations in the populations of *Orthomus berytensis* coming from modern-day Israel.

**Type material. *Feronia elongata* Chaudoir, 1859.** We were unable to find any type specimen(s) of this taxon, but the description and especially the distribution Chaudoir gave for his species (Chaudoir 1859: 116) let us draw the conclusion that he dealt with *Orthomus berytensis*. This view is consistent with that of Chaudoir (1873: 105) who synonymized his *Orthomus elongatus* partly with *Orthomus longulus* sensu Wollaston (1864: 47), and Wollaston (1865: 39), which concerns, according to Machado (1992: 259), *Orthomus berytensis*. On the other side, the first locality listed by the author from among the type one is “Moräa”. Morea was the name of the Peloponnese Peninsula in South Greece during the early modern period (ca. 1500–1800). At present, based on recent data (Arndt et al. 2011: 58, present work: ‘Other material studied’), we certainly know that only *Orthomus berytensis* inhabits this part of the East Mediterranean. Therefore we propose the synonymy of *Feronia elongata* with *Feronia berytensis*.

**Type material. *Orthomus longior* Chaudoir, 1873 (specimens belonging to *Orthomus berytensis*).** The type series in MNHP contains 3 ♀♀ which belong to *Orthomus berytensis*. These samples are considered syntypes of *Orthomus longior* as they and a fourth specimen, which is here designated lectotype of *Orthomus longior* (for that specimen see under *Orthomus longior*), were found alongside in the former collection of René and Charles Oberthür who acquired the Chaudoir collection. Each of the three specimens bears the labels: “*longior*” [red handwritten with ball-pen on white label by Mateu], and, subsequently added: “Paralectotype *Orthomus longior* Chaudoir, 1873 B.Guéorguiev & D.W.Wrase des. 2012” [black print on red label], and: “*Orthomus berytensis* (Reiche & Saulcy) det. B.Guéorguiev” [black print on white label].

##### Other material studied.

**IMPRECISE LOCALITY:** 1 ♂, “Syrie Gory.” (MHNG).

**GREECE:** 1 ♂, “Graecia” / “Sammlung Schroeder” (MIZ); 1 ♀, “L. Miller Graecia” (ZMAN). **Atticí:** 1 ♂, “Graecia Attica”, von Oertzen” (ZMAN); 3 ♂♂, 2 ♀♀, “b. Athen v. O.” (ZMAN); 1 ♂, Peloponnes”, X 2009, Umlauf leg. (DWBG). **Crete:** Rethymno Prefecture, 1 ♀, “Adele, IV.1986, Egger leg.” (NMW).

**NORTHERN CYPRUS: Gazimağusa District:** 2 ♀♀, Agia Napa, 8.IV.1983, J. Nielsen leg (DWBG); 1 ♂, 2 ♀♀, 3 km W Agia Napa, 9.-14.IV.1999, A. Pütz leg. (DWBG); 1 ♀, Agia Napa env., middle IV 1998, M. Sieber leg. (DWBG); 6 ♂♂, 1 ♀, Ydatodexameni Kouklion, Mantres tou Prastio, ca 16 km W Famagusta, 500 m, 35°07'N, 33°46'E (fallow land with Salicornia/Sueda), 20.II.2011, D.W.Wrase leg. [5] (DWBG); 2 ♂♂, same data but: loamy field edge (DWBG).

**UN BUFFER ZONE** (Cyprus): 5 spec., Achna dam, 11.12.2006, under stones, K. Austin & E. Small leg. (BMNH, NMNHS).

**REPUBLIC OF CYPRUS: Larnaca District:** 1 ♂, 2 ♀♀, Pyla [“Pula”], 22.I.1992, J. Hořyna leg. (DWBG).

**SYRIA: Gouvernement Dar'a:** 1 ♀, Buṣrā (free zone), 100 km S Damascus, 5.XII.2007, R. Kmeco leg. (DWBG).

**ISRAEL:** 1 ♂, Palestine Negev, 8.2.19, leg. Bythinsky-Salz (TAU); 5 ♂♂, 5 ♀♀, Palestyna Dzebata 25.XII.25 (MIZ). - **Jerusalem District:** 1 ♂, Kiryat Anavim, 8.4.1948 (TAU). **Northern District:** 1 spec., Golan, Tel Quazir, 29.3.-26.4.87, Richter leg. (NMW). - **Tel Aviv District:** 1 ♂, Tel-Aviv, 19.X.1987, G. Coulon leg. (TAU). - **Central District:** 1 ♂, 1 ♀, SW Khadera (brackish pond), 27.III.2008, D.W.Wrase leg. (DWBG); 2 ♂♂, 1 ♀, Nitsanim, dunes betw. Ashdod and Ashkelon, 29.III.2008, D.W.Wrase leg. (DWBG). - **Haifa District:** 2 ♂♂, 1 ♀, Atlit, 16.XI.1998, V. Chikatunov leg. (TAU); 2 ♀♀, Salines at Atlit, S Haifa, 30.III.2008, D.W.Wrase leg. (DWBG). - **South District:** 1 ♀, Revivim, 2.8.1958, Coll. Krystal J. (TAU); 1 ♀, Segula, Qiryat Gat, 5.v.1996, V. Chikatunov leg. (TAU); 1 ♀, Ashdod, 7.12.96, R. Hoffman leg. (TAU); 1 ♂, 1 ♀, Ofaqim, 11.ii.1997, L. Friedman leg. (TAU); 2 ♂♂, 1 ♀, Ha Bsor, Nakhal Bsor, ca 12 km SW Ofakim (banks, leaf litter, under plants, sifted), 22.III.2008, D.W.Wrase leg. (DWBG); 1 ♂, Gilat, SW Jerusalem, E Gaza, 31°20'N, 34°40'E, olive plantation, 29.III.2011, M. Kurtz leg. (MPHG).

**Gaza Strip:** 8 ♂♂, 5 ♀♀, Env. Gaza, 30.III.1987, W. Heinz leg. (DWBG); 9 spec., Gaza Umg., 29.3.–26.4.87, Richter leg. (NMW).

**LIBYA: Tripoli District:** 8 spec., Tripoli, 0-20 m, 12-18.04.1998, P. Beron leg. (NMNHS); 1 ♀, Tripoli, IV 1982, Březina leg. (DWBG); 4 spec., Tripoli, Soani Road, 4.XII.1982, V. Pamukov leg. (NMNHS); 1 ♀, 4 km W Sidi Muhammad al Mabkhud, 32°28'53.3"N, 20°56'29.4"E, 333 m, 21.V.2002, A. Reiter leg. (DWBG). - **Binghazi District:** 1 ♂, 1 ♀, Tolmeitha (Ptolemais), 19.III.2000, J. Kny & P. Rudich leg. (DWBG). - **Jabal al Akhdar District:** 1 ♂, Susa, Apollonia, 22.II.83, O. Kodym leg. (DWBG).

**EGYPT:** 2 ♀♀, Cairo-Alexandria, 8.5.1956, W. Kühnell leg. (NMW). - **Faiyum Governorate:** 4 ♂♂, 2 ♀♀, Kom Oshim, 1.5.1956, W. Kühnell leg. (NMW). - **North Sinai Governorate:** 1 ♀, El-Arish 11.1.57, A. Sabay leg. (TAU). - **Alexandria Governorate:** 1 ♂, 100 km S Alexandria, 19.I.1994, W. Ullrich leg. (DWBG). - **Beni Suef Governorate:** 1 ♂, 1 ♀, El Shanawaya, 1.IX.1994, W. Ullrich leg. (DWBG); 1 ♂, the same but 29.V.1995 (DWBG); 1 ♀, the same but 4.VI.1995 (DWBG); 5 ♂♂, 3 ♀♀, the same but 12.V.1995 (DWBG). - **Cairo Governorate:** 1 ♂, 1 ♀, Helwan, 19.I.1931, W. Roszkowski leg. (MIZ); 1 ♀, El Maadi, 8.XI.1993, W. Ullrich leg. (DWBG). - **Ismaïlia Governorate:** 3 ♀♀, Abu Suweir-el-Mahatta, 19.XII.1995, W. Ullrich leg. (DWBG); 1 ♂, Waraura, 4.VI.1995, W. Ullrich leg. (DWBG).

##### Distribution in eastern Mediterranean area.

Reported from Greece: Greek mainland (“Attika” [Attica], Ionian Island (“Zante [Zakynthos]), Apfelbeck 1904: 257, as *Pterostichus barbarus*, and Aegean Islands (“Rodos” [Rhodes]), Schatzmayr 1935: 242, as *Pterostichus barbarus*, 1942: 67, as *Pterostichus barbarus longulus*). In Greece recently collected only on Crete and also on the Peloponnese. Turkey (Casale and Vigna Taglianti 1999: 382), Syria (only in the extreme South West), Israel, Gaza Strip, on Cyprus, in Libya (coastal regions), and Egypt (mainly in the North West).

#### Orthomus longior

Taxon classificationAnimaliaColeopteraCarabidae

Chaudoir, 1873

Figs 2 , 5 , 9 , 13 , 17

Othomus [sic!] *longior* Chaudoir, 1873: 105 (type locality: “Sidon”), (as locality of LT), part
Feronia (Argutor) longula Reiche & Saulcy, 1855: 616, part
Orthomus sidonicus Chaudoir, 1873: 110 (type locality: “Sidon (Syrie)” [Saïda, Lebanon], syn. n.
Orthomus longulus sidonicus : Mateu, 1955: 56, 63

##### Type material.

***Orthomus longior* Chaudoir, 1873 (specimens belonging to *Orthomus longior*).** Consists of 4 ♀♀ preserved in MNHP, investigation revealed non-conspecificity. Three specimens are identical with the lectotype of *Orthomus berytensis* (for these specimens see under *Orthomus berytensis*). The fourth female possesses labels: “Sidon” [handwritten on white label by Chaudoir], “*longior*” [handwritten in red with ball-pen on white label by Mateu]. This specimen is conspecific with the holotype of *Orthomus sidonicus*. As only that bears a locality label we choose this one as lectotype of *Orthomus longior*. This action led to the synonymy of *Orthomus longior* with *Orthomus sidonicus*. The two names are published on the same date in the same work (Chaudoir 1873). As “first reviewing authors”, we give precedence of the former following the Article 24.2.2 of the Code (ICZN 1999). Hence, the specimen in question is supplied with additional label: “Lectotype *Orthomus longior* Chaudoir, 1873 B.Guéorguiev & D.W.Wrase des. 2012” [black print on red label].

**Type material. *Feronia longula* Reiche & Saulcy, 1855 (specimens belonging to *Orthomus longior*).** The following three male and one female specimen belong to the type series of *Feronia longula* (MHNG, see below). The study of the genitalia of all males, as well as the external characters of both sexes proved that all specimens belong to *Orthomus longior*: 1 ♂, “Beyrouth” [Reiche's handwriting on yellow label], “Coll. Reiche” [black print on white label], “*Orthomus longulus* Reiche Label MHNG 2010” [black print on white label by G. Cuccodoro]; 1 ♂, “*longulus*” [Reiche's handwriting on white label], “Coll. Reiche” [black print on white label], “*Orthomus longulus* Reiche ‘Egypte, Syrie.’ Label MHNG 2010” [black print on white label by G. Cuccodoro]; 1 ♂, “Coll. Reiche” [black print on white label], “*Orthomus longulus* Reiche ‘Egypte, Syrie.’ Label MHNG 2010” [black print on white label by G. Cuccodoro]; 1 ♀, “Nazareth” [Reiche's handwriting on yellow label], “Coll. Reiche” [black print on white label], “*Orthomus longulus* Reiche Label MHNG 2010” [black print on white label by Cuccodoro]. All specimens with labels, subsequently added: “Paralectotype *Feronia longula* Reiche & Saulcy, 1855 B.Guéorguiev & D.W.Wrase des. 2012” [black print on red label], “*Orthomus longior* Chaudoir, 1873 det. B.Guéorguiev” [black print on white label].

**Type material. *Orthomus sidonicus* Chaudoir, 1873.** Holotype ♂ in MNHP, with extracted genitalia. The median lobe and parameres are well preserved and glued on a separate card pinned beneath the specimen. The specimen bears the following labels: “Sidon.” [handwritten on white label by Chaudoir], “*sidonicus* Chaud.” [handwritten in red with ball-pen on white label by Mateu], and, subsequently added: “Holotype *Orthomus sidonicus* Chaudoir, 1873” [black print on red label by Guéorguiev], “*Orthomus longior* Chaudoir, 1873 det. B. Guéorguiev” [black print on white label].

##### Other material studied.

**ISRAEL: Northern District:** 2 ♂♂, 2 ♀♀, Upper Galilee, Nahal Kziv, 30.i.1999 / 6.iii.1999, M. Finkel (TAU); 1 ♂, 1 ♀, Upper Galilee, Meron Mts., Meron Field School, ca 1000 m (open woodland), 8.–20.III.2008, D.W.Wrase leg. (DWBG); 1 ♂, 1 ♀, Upper Galilee, Meron Mts., Har Meron, 850 m (cedar/pine forest, pitfall trap), 6.V.1996, P. Schnitter & K. Staven leg. (DWBG); 1 ♂, Upper Galilee, Meron Mts., Nakhar (Wadi) Moran, 1 km W Meron field school, ca 900 m (N. slope, slope spring, under stones), 11.III.2008, D.W.Wrase leg. (DWBG); 1 ♀, Upper Galilee, Meron Mts., Har Meron, Kamin Rom, 1100 m, 32°59.447'N, 035°24.669'E (open stony grazing land, limestone), 1.IV.2008 D.W.Wrase leg. (DWBG); 1 ♂, 1 ♀, Upper Galilee, Ya’ar Bar’am, ca 1.5 km W Jish (Gush Khalav), ca. 700 m (edge of oak forest), 9.III.2008, D.W.Wrase leg. (DWBG); 1 ♂, N. Golan Heights, Qalat Nimrod, 300–600 m 7.IV.1985, W. Heinz leg. (DWBG); 1 ♂, Golan Heights 19.iv.1994, M. Warburg leg. (TAU); 2 ♂♂, 1 ♀, Golan Heights, Mas’ada, Ya’ar Odem Reserve, 934 m, 33°13.449'N, 035°45.184'E (grazing woodland, oaks, litter sifted), 21.IV.2006, D.W.Wrase leg. (DWBG); 1 ♂, 1 ♀, Golan Heights, Ya’ar Odem S Mas’ada, 33°13.449'N, 035°45.184'E, 934 m (*Quercus boissieri*/*calliprinos* forest, under stones), 10.III.2008, D.W.Wrase leg. (DWBG). - **Haifa District:** 1 ♀, Haifa [“Syrien Haifa Reitter”] (NMW); Carmel Ridge: 1 ♀, Nahal Oren, Mt. Carmel, 15.11.1995, Pavlicek & Chikatunov leg. (TAU); 3 ♂♂, 4 ♀♀, ‘En Ya’aqov, 23.iii.2006 / 8.ii.2007 / 19.iii.2007, I. Schtirberg’ (TAU).

**LEBANON: Muhāfazat Bayrūt:** 2 ♀♀, Beirut [“Beyruth, Syr. coll. Plason” / “*sidonicus* Chd. det. Ing. Jedlička”] (MIZ). 1 ♂, 3 ♀♀, E Bayrūt, Faytroun, 34°.00'N, 35°44'E, ca. 1100 m, 30.X.2012, Chr. Reuter leg. (CRHG, DWBG). 14 ♂♂, 7 ♀♀, S Bayrūt, Dammour env., ca. 200 m, pitfall trap, II 2013, Chr. Reuter leg. (CRHG, DWBG, JSAG, NMNHS). - **Chouf District:** 1 ♀, Barouk, Mount Lebanon, El Mir massif, 1700-1950 m, 20.V.2006, T. Tichý leg. (DWBG.). - **Keserwan District:** 4 ♀♀, Balloun at Jitra, S Jounié, 600 m, 9./14.IV.1997, W. Heinz leg. (DWBG). 10 ♀♀, Rayfoun, ca. 33°58'N, 35°42'E, mixed decidous forest, 800–900 m, 18.XI.2012, Chr. Reuter leg. (CRHG, DWBG, JSAG, NMNHS). 1 ♂, 3 ♀♀, same data but: mixed oak forest, ca. 990 m, 15.III.2013 (CRHG, DWBG). 1 ♂, 1 ♀, same data but: 30.III.–15.IV.2013 (CRHG, DWBG).

**SYRIA: Al-Lādhiqīyah:** 2 ♀♀, Lattaki-Slenfe, 27.4.1990, Reuter leg. (NMW); 1 ♂, Şlinfah, Abal an Nusayriah Mt., 1200 m, 24.–26.V.1995, P. Kabátek leg. (DWBG); 2 ♂♂, 5 ♀♀, Jabāl Ansarya, At Tammāzah, 790 m, 34.15.404N, 030.10.136E, 20.XII.2006, R. Sehnal leg. (DWBG); 1 ♀, Slenfeh, 18.4.2010, Vl. Skoupý leg. (VSKC).

**TURKEY: Antalya Province:** 1 ♀, Alanya-Yayla, 1000 m, 13.5.1987, Steiner leg. (NMW); 1 ♀, Manavgat, Kiselot, 3.1.91, 10 m HN, Wunderle leg. (NMW); 1 ♂, Antalya env., 8.II.1999, J. Blümel leg. (DWBG); 1 ♂, 1 ♀, Avsallar near Incekum beach, 22 km W Alanya, 9.-23.V.1995, A. Pütz leg. (DWBG); 2 ♀♀, Incekum, env. Avsallar, under stone, II 1999, Schlarbaum leg. (TFPG); 3 ♂♂, 1 ♀, Gedevit-Yayla near Alanya, ca 1100 m, 10.IV.1992, W. Heinz leg (DWBG, JSAG); 1 ♂, Karaburu near Alanya, middle V 1997, M. Sieber leg. (DWBG); 1 ♂, Manavgat env., 3.I.1991, V. Assing leg. (DWBG); 1 ♀, E. Taurus Mts., Çaltepe env. (Manavgat District), 1600 m, 37.18N, 31.12E (subalpine), 10.–14.VI.2004, P. Croy leg. (DWBG). - **Hatay Province:** 1 ♀, W Yayladaği, 475 m, 35°54'30.8"N, 36°01'11.3"E, 05.-10.05.2006, Schnitter leg. (PSHG).

##### Wrong locality.

1 ♀, Amasya: Amasya [“Amasia coll. Kraatz”] (DWBG).

##### Male genitalia

(15 specimens examined).

##### Distribution.

Turkey (only Antalya and Hatay Province), Syria (only Latakia Governorate), Lebanon (several coastal districts), North Israel (Northern District; Haifa District). First species records to Turkey and Syria.

#### Orthomus longulus

Taxon classificationAnimaliaColeopteraCarabidae

(Reiche & Saulcy, 1855)

Figs 3 , 6 , 10 , 14

Feronia (Argutor) longula Reiche & Saulcy, 1855: 616 (type locality: “De Beyrouth”), part
Feronia (Argutor) berytensis Reiche & Saulcy, 1855: 618, part
Orthomus longior Chaudoir, 1873: 105, part
Orthomus longulus s.str.: Mateu 1955: 56, 60

##### Type material.

***Feronia longula* Reiche & Saulcy, 1855 (specimens belonging to *Orthomus longulus*).** The type series of *Feronia longula* consists of 11 syntypes, 4 ♂♂, 6 ♀♀ of them in MHNG and 1 ♂ in MNHP. The specimens in MHNG are placed under a Melly’s taxa label “*longulus* Reiche., Egypte, Syrie” [handwritten by Melly with pen] pinned to the bottom of the drawer, which thus are to be considered as pertaining collectively to all specimens. Our revision revealed that the specimens in MHNG are not conspecific but belong to three distinct species. Three males and one female belong to *Orthomus longior* (for these specimens see under *Orthomus longior*), while another male and another female belong to *Orthomus berytensis* (for these specimens see under *Orthomus berytensis*). Only four females are representatives of *Orthomus longulus*, labelled individually as follow. One female selected for lectotype: “…” [handwritten remnant on small quadratic yellow label], “Beyrouth” [Reiche's handwriting on brown label], “Coll. Reiche” [black print on white label], “*Orthomus longulus* Reiche Label MHNG 2010” [black print on white label by Cuccodoro], “Lectotype *Feronia longula* Reiche & Saulcy, 1855 B.Guéorguiev & D.W.Wrase des. 2012” [black print on red label]; 3 ♀♀, “Coll. Reiche” [black print on white label], “*Orthomus longulus* Reiche ‘Egypte, Syrie.’ Label MHNG 2010” [black print on white label by Cuccodoro], “Paralectotype *Feronia longula* Reiche & Saulcy, 1855 B.Guéorguiev & D.W.Wrase des. 2012” [black print on red label]. The specimen from MNHP is a male, with previously extracted genitalia and glued to a separate card pinned beneath the specimen. The most part of its aedeagus is destroyed, certainly by a species of the genus *Anthrenus* Geoffroy, 1762, but the apical lamella is still preserved. This male is conspecific with the above last four females from MHNG and it is designated as paralectotype, too: “*longulus* type Reiche” [handwritten on white label by Mateu], “Paralectotype *Feronia longula* Reiche & Saulcy, 1855 B.Guéorguiev & D.W.Wrase des. 2012” [black print on red label]. All five specimens, pertaining to the true *longulus*, with a label, subsequently added: “*Orthomus longulus* (Reiche & Saulcy) det. B.Guéorguiev” [black print on white label].

##### Other material studied.

**ISRAEL:** - **Northern District:** 1 ♂, “Palestine. …Galilee XII.1924 O. Theodor.” (BMNH); 1 spec., “Nazaret, 17.3.-3.4.87, Kfar …. L. Blumenthal leg.” (NMW); 3 ♂♂, 5 ♀♀, Megiddo, Ein Ha’emek, 230 m, Getreideacker, 08.V.1996, Schnitter & Staven leg. (DWBG); 1 ♂, 4 ♀♀, “Merom Golan, 12.VI.2000, V. Chikatunov leg.” (TAU); 2 ♂♂, 3 ♀♀, Upper Galilee, N. sea shore of Sea of Galilee, Ein Sheva (Tabkha), -192 m, 32°52.453'N, 035°32.726'E (stony and loamy pasture), 25.IV.2006, D.W.Wrase leg. (DWBG); 1 ♂, Lower Galilee, ca 4 km W Tamra, (route 70), 32°51.799'N, 035°10.292'E (loamy field edge), 25 m, 25.IV.2006 D.W.Wrase leg. (DWBG); 1 ♂, 6 ♀♀, Upper Galilee, Ha Khula Valley, Ma’agar Einan lake, 73 m, 33°05.137'N, 035°34.730'E (toe of dam, in moist loamy soil), 1./2.V.2006 D.W.Wrase leg. (DWBG, JSAG); 1 ♂, Bir el Maksur, 32°45.901'N, 035°13.883'E, 23.II.2005, W. Starke leg. (DWBG); 1 ♀, Nazareth, Kfar ?Hochbreeh, 17.III.-3.IV.1987, Blumenthal leg. (DWBG). - **Haifa District:** 2 ♂♂, 2 ♀♀, Haifa [“Syrien Haifa Reitter”] (BMNH, MHNG, MIZ, NMW); 1 ♀, Mount Carmel, 23.XII.25 (MIZ); 4 ♀♀, Haifa, 15.XII.1941 / 8.I.1942 / 4.XII.1954 Bytinski-Salz (TAU); 1 ♀, Nahal Oren, Mount Carmel, 18.3.1996, Pavliček & Chikatunov leg. (TAU); 1 ♀, Haifa, Check Post, 8.II.2000, V. Chikatunov & T. Pavliček leg. (TAU); 1 ♂, Mount Carmel, Ya’ar ha- Ya’aramin ca. 500 m (under stones), 30.III.2008, D.W.Wrase leg. (DWBG).

##### Wrong locality.

1 ♂, “42 St.” / “*Orthomus berytensis* Reich. Portugal Dr Stierlin (above), 2. b. (underneath)” (MHNG).

##### Male genitalia

(9 specimens examined).

##### Distribution.

North Israel (Northern District; Haifa District), Lebanon (Beyrouth, type material).

### Key to the East Mediterranean species of *Orthomus* Chaudoir

**Table:** 

1	Abdominal sternites densely and deeply punctured and rugose laterally	2
–	Abdominal sternites superficially punctured or smooth laterally	3
2	Pronotum with hind angles obtuse at tip, often with small denticles protruding laterally (Fig. 16). All discal setiferous punctures of elytra as a rule situated close to or in stria 3. Elytral striae smooth, sometimes with a shallow punctuation. Mesepisternum smooth or with shallow punctuation only. Elytral microsculpture in females consisting of isodiametric meshes almost regularly arranged (as in *Orthomus longior*). Median lobe of aedeagus toward apex distinctly shifted to the left, apical lamella narrowed distally, almost round at tip (dorsal aspect) (Figs 4, 8).	*Orthomus berytensis* (Reiche & Saulcy, 1855)
–	Pronotum with hind angles almost right-angled, rounded at tip (Fig. 17). Second discal setiferous punctures of elytra mostly adjoining stria 2 (rarely one or two punctures in the middle of interval 3; by exception one puncture adjoining stria 3). Elytral striae ± strongly punctured throughout. Mesepisternum with distinct, dense and coarse punctuation. Elytral microsculpture in females somewhat irregular, consisting of isodiametric meshes mixed with little transverse meshes. Apical lamella of median lobe with a single angle at left side, right side rounded (dorsal aspect) (Figs 6, 10)	*Orthomus longulus* (Reiche & Saulcy, 1855)
3	Elytral striae smooth, sometimes with a weak punctuation laterally and apically, situation of elytral discal punctures as in *Orthomus longulus*. Elytral microsculpture in females consisting of isodiametric meshes almost regularly arranged (as in *Orthomus berytensis*). Pronotum with hind angles almost right-angled at tip, with small denticles protruding laterally, similar to *Orthomus berytensis* (populations from Turkey) or with hind angles somewhat obtuse-angled, rounded at tip, as in *Orthomus longulus*, rarely with suggestion of a denticle (populations from Lebanon, Syria, Israel). Apical lamella of median lobe angled at both sides (dorsal aspect) (Figs 5, 9)	*Orthomus longior* Chaudoir, 1873

### II. Notes on West Mediterranean *Orthomus* species

#### Orthomus velocissimus akbensis

Taxon classificationAnimaliaColeopteraCarabidae

Mateu, 1955

Figs 7 , 11 , 15

Orthomus barbarus akbensis Mateu, 1955: 57, 74 (type locality: “Akbes, Siria”, patria falsa)
Feronia hesperica ? Motschulsky, 1849: 73 (type locality: “le midi de l’Espagne”)
Orthomus barbarus expansus form. *transiens* Mateu, 1957: 99, unavailable
Orthomus barbarus expansus form. *malacensis* Mateu, 1957: 99, unavailable
Orthomus barbarus andalusiacus Mateu, 1957: 103 (type locality: “Prov. de Málaga: Gobantes”), syn. n.
Orthomus expansus malacensis Jeanne, 1981: 45 (type locality: “Málaga”)

##### Type material.

***Orthomus barbarus akbensis* Mateu, 1955.** Holotype ♂ (IRSNB): “Holotypo” [print on red label], “Syrie Akbes” [print on white label], “*Orthomus barbarus* subsp. *akbesensis* [sic!] mihi J. Mateu det., 1951” [mixed handwritten & print on white label]. Genitalia extracted, well preserved, glued to a separate card pinned beneath the specimen.

##### Other material studied.

**PORTUGAL:** - **Faro:** 1 ♂, Lagos env. 27./28.X.2006, V. Neuman leg. (cWR). 1 ♀, E Bensafrim, N Lagos, 28.III.1995, Chr. Bayer leg. (cWR). 1 ♀, Sagres, 4.IV.1989, M. Sachez (cWR). 1 ♂, 1 ♀, Sagres, 1 km from coast, 9.IV.1992, V. & C. Neumann leg. (cWR). 1 ♂, Sierra Monchique, Cabo de Sáo Vicente, 11.V.1992, V. & C. Neumann leg. (cWR). 1 ♂, 2 ♀♀, Carvoeiro, 10.V.1997, B. Nickel leg. (cWR).

**SPAIN:** - **Córdoba:** 1 ♂, Córdoba, escuela, 10.IV.2003, T. Tichý leg. (cWR). - **Granada:** 1 ♂, La Herradura, 27.XII.1998-3.I.1999, G. Siering leg. (cWR). - **Málaga:** 1 ♂, Malaga, 28.VIII.1996, P. Beron leg. (NMNHS). 1 ♀, “Sierra de Tejeda, Competa S, 600 m, 6.4.2001, Ch. Bauer leg.” (NMNHS).

##### Remarks.

*Orthomus berytensis akbensis* was described from “Akbes, Siria”, based on a male and a female specimen. Mateu characterized it as having the pronotal base not bordered bilaterally, the pronotum widest at about middle, and with basal fovea punctured, the elytral striae hardly punctured, the metatibia in male crenulate at internal side, and the median lobe (Mateu 1955: 75, Fig. 9) somewhat differing from *Orthomus barbarus berytensis*.

The study of the structure of the median lobe of aedeagus and the parameres, as well as selected external features in the holotype of *Orthomus barbarus akbensis* demonstrated that it is really different from the other three East Mediterranean species. Its comparison with various taxa of the genus revealed that it is identical with specimens of *Orthomus velocissimus andalusiacus* Mateu, 1957 (compare Figs 4, 8, 12 and Mateu 1957, Lámina IV, Figs 5–8, Pupier and Coulon 2013: 224, Fig. 1d). Hence, we synonymize the latter with *Orthomus barbarus akbensis* which becomes the senior synonym and therefore the name of a valid subspecies of *Orthomus velocissimus* (Waltl, 1835).

Lorenz (2005: 265) formally declared *Feronia hesperica* as a *nomen oblitum*, in spite of the fact that a junior name has never been declared as *nomen protectum*. This statement is incorrect, since after 1899 this taxon was cited at least twice in the coleopterological literature. Heyden et al. (1906: 86) and Csiki (1930: 612) recorded it as synonym of *Pterostichus (Orthomus) barbarus*. The type locality cannot be fixed exactly geographically, Motschulsky (1849: 52) wrote that the coleopterological yields the collector Handschuh made in 1847 in southern Spain came from “principalement aux environs de Carthagène“, which means that other parts of southern Spain cannot be excluded. Thus, we accept the view of Mateu (1957: 103) and list *Feronia hesperica* as a questionable senior synonym of *Orthomus velocissimus akbensis*, before the identity of the former can be settled.

The name *malacensis* Mateu was used as an infrasubspecific one (as also *transiens*) and is therefore not available according to Article 45.5 of the Code (ICZN 1999). The name *malacensis* Jeanne is the available name for *malacensis* Mateu (see also Serrano 2003: 45), adopted by Jeanne (1981) in agreement with Art. 45.5.1.

Lorenz (1998: 249, 2005: 265) combines *Orthomus malacensis* Jeanne with the year 1978. Though Jeanne’s work is part of “Tome VIII, 1978-1980” of the Bulletin de la Société linnéenne de Bordeaux, it was not printed until 1981 (see last page of that paper).

The application of Art. 72.4.4 specifies the type locality of *Orthomus malacensis* Jeanne.

### Updated Checklist of the species of *Orthomus* Chaudoir, 1838

1. *abacoides* Lucas, 1846: 46 (*Oodes*) – Algeria

= *trapezicollis* Chaudoir, 1859: 117 (*Feronia*)

= *occidentalis* Gautier des Cottes, 1870: 299

= *modestus* Reiche, 1871: 427 [replacement name]

Note1: See Note2 under *Orthomus barbarus*.

2. *achilles* Wrase & Jeanne, 2005: 888 – Algeria

3. *aquila* Coquerel, 1859: 768 (*Feronia*) – Algeria

= *numidus* Chaudoir, 1859: 118 (*Feronia*)

4. *aubryi* Jeanne, 1974: 68 – Spain

5. *balearicus* Piochard de la Brûlerie, 1868: lxxx (*Feronia*) – Balearic Islands

= *szekessyi* Jedlička, 1956: 392 (*Pterostichus*) syn. n.

6.1. *barbarus barbarus* Dejean, 1828: 261 (*Feronia*) – Portugal, Spain, France

? *rectangulus* Fairmaire, 1859: li (*Feronia*)

= *expansus* Mateu, 1957: 98

= *logronicus* Mateu, 1957: 98 [unav.]

6.2. *barbarus formenterrae* Breit, 1933: 67 (*Pterostichus*) – Balearic Islands

6.3. *barbarus penibeticus* Mateu & Colas, 1954: 53 – Spain

Note1: *Feronia rectangulus* described from Batna, Algeria, probably is to fall in synonymy with another species of *Orthomus*, rather than with *Orthomus barbarus*.

Note2: Pupier and Coulon (2013: 217, 218) say that, according to Zaballos and Jeanne (1994) and Ortuño (1996), *formenterrae* and *penibeticus* are subspecies to *Orthomus abacoides*. The contrary is the view of Serrano (2003) and Bousquet (2003) who treat these taxa as subspecies of *Orthomus barbarus*. The last reviewing authors (Pupier and Coulon 2013: 218, 219, 223) say: “Il en est de même de la subordination de *penibeticus* et *formenterrae* en tant que sous-espèces d'*Orthomus abacoides*”, but also: “Cependant l’incertitude du rattachement de ces formes à *Orthomus barbarus* tel que le préconisent Serrano (2003) et Bousquet ne permet pas, sans etude suplementaire avec suffisamment de materiel, de fixer leur status.” Due to these uncertainties we follow the view of Serrano (2003).

Note3: *Pterostichus (Orthomus) szekessyi* Jedlička, 1956 was described from the Balearic Islands, based on a male without an exact locality (Jedlička, 1956). The holotype is stored in the collections of the Magyar Természettudományi Múzeum, Budapest and one of us (DWW) has examined it. It is in fairly good condition, the right last four antennomeres and the left metatarsus are lacking. The specimen was originally pinned and subsequently glued to card, with aedeagus not extracted (now it is glued to a separate card beneath the specimen). It is labelled with: “Balearen” [handwritten on white label], “Typus” [black print on red label], “Pterostichus (Orthomus) Sze’kessyi sp.n. det. ING. JEDLIČKA” [red label, species name handwritten in black by Jedlička, the rest printed]. It agrees in all characters, including the construction of the median lobe of the aedeagus, with these ones of *Orthomus balearicus*. Hence, we propose the synonymy of *Pterostichus (Orthomus) szekessyi* with *Feronia balearicus* Piochard de la Brûlerie, 1868. The specimen is additionally labelled with “Orthomus balearicus PIOCHARD DE LA BRȖLERIE, 1868 D.W. Wrase det. 2014” [black print on white label].

7. *berytensis* Reiche & Saulcy, 1855: 618 (*Feronia*) – Sardinia, Sicily, Malta

= *proelongus* Reiche & Saulcy, 1855: 619 (*Feronia*) syn. n. – Greece, Turkey, Cyprus

= *elongatus* Chaudoir, 1859: 116 (*Feronia*) syn. n. – Syria, Lebanon, Israel

= *haligena* Wollaston, 1860: 87 (*Pterostichus*) – Canary Islands, Morocco

? *varinii* Gautier des Cottes, 1866: 178 (*Feronia*) – Tunisia, Libya, Egypt

= *atlanticus* Fairmaire, 1875: 543 (*Feronia*)

= *oceanicus* Mateu, 1951: 283 [unav.]

= *muluyensis* Antoine, 1957: 205

Note1: *Feronia varinii* described from Sardinia, is most probably a synonym of *Orthomus berytensis* rather than of *Orthomus barbarus*.

8.1. *dimorphus antoinei* Mateu, 1955: 70 – Morocco

8.2. *dimorphus dimorphus* Antoine, 1933: 85 – Morocco

9. *discors* Wollaston, 1864: 47 (*Pterostichus*) – Canary Islands

= *persimilis* Harold Lindberg, 1950: 2 (*Pterostichus*)

10. *hispanicus Dejean*, 1828: 260 (*Feronia*) – Spain

= *quadrifoveolatus* Chaudoir, 1859: 117 (*Feronia*)

11.1. *lacouri haroldi* Pupier & Coulon, 2013: 221 – Morocco

11.2. *lacouri kocheri* Mateu, 1955: 68 – Morocco

11.3. *lacouri lacouri* Antoine, 1941: 38 (*Platysma*) – Morocco, Algeria

11.4. *lacouri pupieri* Jeanne, 1988: 12 – Algeria

12. *leprieuri* Pic, 1894: 104 – Algeria, Tunisia

13. *longior* Chaudoir, 1873: 105 – Turkey, Syria, Lebanon

= *sidonicus* Chaudoir, 1873: 110 syn. n. – Israel

14. *longulus* Reiche & Saulcy, 1855: 616 (*Feronia*) – Lebanon, Israel

15. *maroccanus* Chaudoir, 1873: 108 – Spain, Morocco

= *humeralis* Antoine, 1957: 208 [unav.]

16. *perezii* Martínez & Saez, 1873: 57 (*Feronia*) – Spain

17. *planidorsis* Fairmaire, 1872: 420 (*Feronia*) – Spain, France

18. *poggii* Leo & Magrini, 2002: 510 – Italy (Isola il Toro)

19. *rubicundus* Coquerel, 1859: 769 (*Feronia*) – Algeria, Tunisia

= *modicus* Coquerel, 1859: 770 (*Feronia*)

= *manogramma* Chaudoir, 1859: 119 (*Feronia*)

= *minutus* Reiche, 1871: 427

20. *starkei* Wrase & Jeanne, 2005: 882 – Morocco

21.1. *tazekensis rifensis* Wrase & Jeanne, 2005: 885 – Morocco

21.2. *tazekensis tazekensis* Antoine, 1941: 411 (*Platysma*) – Morocco

= *scutellaris* Antoine, 1941: 412 [unav.]

22.1. *velocissimus akbensis* Mateu, 1955: 74 – Spain

? *hesperica* Motschulsky, 1849: 73 (*Feronia*)

= *transiens* Mateu, 1957: 99 [unav.]

= *malacensis* Mateu, 1957: 99 [unav.]

= *andalusiacus* Mateu, 1957: 103 syn. n.

= *malacensis* Jeanne, 1981: 45

22.2. *velocissimus pardoi* Mateu, 1957: 102 – Spain

22.3. *velocissimus velocissimus* Waltl, 1835: 53 (*Argutor*) – Spain

Note1: For *Feronia hesperica* see remarks under *Orthomus velosissimus akbensis*.

### III. Notes on Afrotropical Euchroina

#### Parorthomus gen. n.

Taxon classificationAnimaliaColeopteraCarabidae

http://zoobank.org/1F0CC178-2E35-4E59-8BAC-8E73F1983095

##### Type species.

*Parorthomus socotranus* sp. n.

##### Diagnosis.

A Euchroina genus of beetles that are medium-sized (8.2-10 mm), black coloured, brachypterous, with the following combination of characters: convex eyes; mentum with bifid tooth and large labial pits; pronotum sides posteriorly straight to slightly convex; elytra with 3-4 (rarely 2 or 5) discal setiferous puncture in stria 3/interval 3, with last puncture in posterior third of elytron; intercoxal process of prothorax subquadrate, distinctly bordered at sides and backwards; metaepisterna as wide as long; abdominal sternites V–VII with transverse basal sulci complete and well-impressed; mesotibia and metatibia straight in both sexes, mesotibia distally with slight inner callus in males; tarsomeres glabrous dorsally, with segment 5 setose ventrally; distal part of median lobe of aedeagus considerably curved to left in dorsal aspect; spermatheca with appended gland spherical and elongate diverticulum.

##### Description.

None required because the genus is monobasic, and its characters are the same as those of its type species.

##### Etymology.

A prefix in apposition (masculine), formed from the Greek *παρά*-, meaning “beside”, “near”, “alongside”, and the name of the Mediterranean pterostichine genus *Orthomus* to which the new taxon is related.

#### Parorthomus socotranus sp. n.

Taxon classificationAnimaliaColeopteraCarabidae

http://zoobank.org/3D5BF7E1-9EB4-47FB-92C5-7E2C7D958916

Figs 18 – 24 , 28 , Table 1

Orthomus sp.: Wranik 2003: 442, plate 170, Fig. f.

##### Type material.

Holotype ♂, “Yemen, Socotra Isl., Fimihin, GPS 12.474N, 54.015E, 530 m, x.2000, leg. V. Bejček & K. Št’astný” (DWBG) / “HOLOTYPE *Parorthomus socotranus* sp. n. Guéorguiev, Wrase & Farkač des. 2014” [black print on red label, black framed]. Paratypes 24 ♂♂, 29 ♀♀, labelled as follow: 4 ♂♂, 4 ♀♀, with the same data as the holotype (DWBG, JFPC, JSAG, NMNHS); 6 ♂♂, 10 ♀♀, “Soqotra-Archipel: Soqotra Hoq, Küstenebene bis Höhleneing., Kalk mit einigen Granitfelsen, dichte Veg., 50–320 m 12°36'N, 54°21'E, 5.–6.2.1999 leg.: H. Pohl, SOQ 08” (BMNH, DWBG, HLMD, MPHG); 3 ♀♀, “YEMEN: Socotra Isl. Haghier, 4.-8.X.2000 lgt. V. Bejček & K. Št’astný” (JFPC); 2 ♂♂, 1 ♀, “Yemen, Soqotra-Archipel, Soqotra, Wadi Danegan, Barberfallen, 90 m 12°36'59"N, 54°03'48"E, 28.–30.10.2000 leg.: T. VAN HARTEN & H. POHL SOQ 2000/02a” (HDLM); 2 ♂♂, “Yemen, Soqotra-Archipel, Soqotra, Homhil, Quelle mit Ficus, Licht 12°34'13"N, 54°18'32"E/leg. H. Pohl, 29.10.2000/SOQ 2000/13” (HDLM, MPHG); 1 ♂, “Yemen, Soqotra-Archipel, Soqotra, Wadi Danegan, 90 m 12°36'59"N, 54°03'48"E, 30.10.2000 leg.: T. VAN HARTEN” “SOQ 2000/02” (HDLM); 3 ♂♂, 1 ♀, “Yemen, Soqotra Is.; 28.–29.ix.2003 HOMHIL protected area N 12°34'27" E 54°18'32" 364 m [GPS]; Jan Farkač lgt.” / “YEMEN – SOQOTRA 2003 Expedition; Jan Farkač, Petr Kabátek & David Král” (JFPC, NMNHS); 1 ♂, 1 ♀, “Yemen, Socotra Is., WADI AYHAFT, 24.-26.xi.2003, 12°36'38’’N, 53°58'49’’E, 190 m, [GPS], leg. P. Kabátek” / “YEMEN – SOQOTRA 2003 Expedition; Jan Farkač, Petr Kabátek & David Král” (DWBG); 1 ♂, 1 ♀, “Yemen, Soqotra Is., 2.xii.2003, Al Haghier mts. W slopes, skant area 12°35'52"N, 54°00'01"E 1240 m [GPS], D. Král leg.” / “YEMEN – SOQOTRA 2003 Expedition; Jan Farkač, Petr Kabátek & David Král” (NMPC, RFBN); 1 ♂, “Yemen, Soqotra Is., QAAREH (waterfall), Noged plain, 5.-6.xii.2003, 12°20'10"N, 53°27'56"E, 57 m [GPS], leg. P. Kabátek” / “YEMEN – SOQOTRA 2003 Expedition; Jan Farkač, Petr Kabátek & David Král” (DWBG); 1 ♀, “Yemen, Soqotra Is., 6.–7.xii.2003 Noged plain: WADI IREEH N12°23'11", 53°59'47"E, 96 m [GPS]; Jan Farkač lgt.” / “YEMEN – SOQOTRA 2003 Expedition; Jan Farkač, Petr Kabátek & David Král” (DWBG); 2 ♀♀, “Yemen: Socotra Isl., Wadi Ayhaft, lat. +1395751.449, lon +824616.2897, 27-30.10.2007, pitfall traps, F. Pella leg.” (MBAP); 1 ♀, “YEMEN: Socotra Island E 410 m, 3. ii. 2010 N12°29'41", E 54°09'30" L. Purchart & J. Vybíral lgt.” (NMPC); 1 ♀, “YEMEN: Socotra Island E Homhil area, 410-510 m, 12°34'25"N, 54°18'53"E 9–10. ii. 2010 L. Purchart & J. Vybíral lgt.” (NMPC); 1 ♂, “YEMEN: Socotra Island Aloove area, Hassan vill. env. 12°31.2'N, 54°07.4'E, 221 m Jiři Hájek leg. 9–10.xi.2010” (NMPC); 2 ♀♀, “YEMEN: Socotra Island Al Haghier Mts. Scant Mt. env. 12°34.6'N, 54°01.5'E, 1450 m J. Bezděk 12-13.xi.2010” (NMPC); 2 ♂♂, 1 ♀, “YEMEN, Socotra Island Hagher Mts., Scand Mt. env. montane evergreen woodland 16.–18.vi.2012 12°34.6'N, 54°01.5'E, 1450 m” / “SOCOTRA expedition 2012 J. Bezděk, J. Hájek, V. Hula, P. Kment, I. Malenovský, J. Niedobová & L. Purchart leg.” (NMNHS, NMPC). All paratypes with label: “PARATYPE *Parorthomus socotranus* sp. n. Guéorguiev, Wrase & Farkač des. 2014” [black print on red label, black framed].

##### Diagnosis.

**Figure 18.**
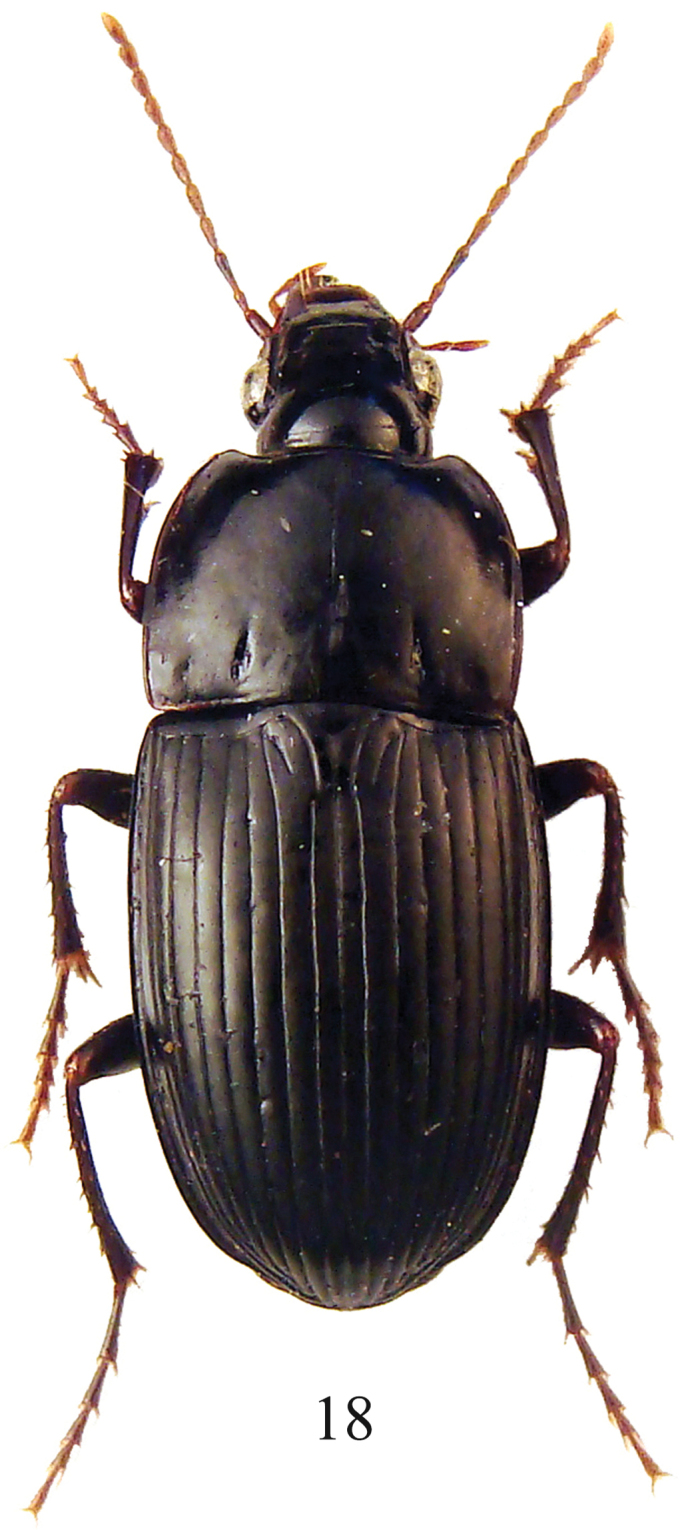
*Parorthomus socotranus* sp. n., female paratype, habitus.

A brachypterous, black coloured species of Euchroina (Fig. 18), with moderately convex, amariform facies, with testaceus appendages, convex eyes, segment 11 of antennae not reaching basal margin of pronotum, elytra with very slight or reduced humeral denticle, elytral interval 3 with three to four (rarely two or five) discal setiferous punctures adjoining stria 3, with last puncture on posterior third of elytron, metaepisterna as long as wide, and median lobe of aedeagus curved to left distally, with apical lamella slightly emarginated at tip.

Values for sizes and ratios among specimens from the type series are shown in Table 1.

**Table 1.** Data on variation in some values among type specimens of *Parorthomus socotranus* sp. n.

type	sex	n	BL/mm	PW/HW	Ø	PW/PL	Ø	PW/PBW	Ø	PL/EL	Ø	EL/EW	Ø
**HT**	♂	1	9.2	1.72	–	1.41	–	1.13	–	0.43	–	1.45	–
**PT**	♂♂	10	8.2–9.6	1.69–1.86	1.77	1.42–1.49	1.46	1.05–1.13	1.10	0.41–0.45	0.43	1.40–1.49	1.44
**PT**	♀♀	12	8.4–10.0	1.68–1.86	1.76	1.43–1.51	1.47	1.05–1.14	1.10	0.41–0.44	0.42	1.37–1.45	1.40

##### Description.

Body length 8.2–10.0 mm (9.2 mm in holotype); width 3.2–3.9 mm (3.6 mm in holotype), maximum width behind the middle of elytra. Head, pronotum, elytra, segments III–IV (in the most cases) of antennae, and ventral surface (without mouthparts) black in mature specimens, light to dark brown in immature specimens; labrum, mandibles, mentum, segments I–II and V–XI of antennae, and sometimes sides of pronotum testaceus; maxillary palpomeres, labial palpomeres, and labium rufous; coxae, femora, and tibiae of legs dark brown or testaceus, trochanters and tarsomeres mostly rufous.

Microsculpture distinct on the whole dorsal and ventral surfaces (including coxae, trochanters and femora), consisting of isodiametric and slight transversal meshes, more apparent in females (female specimens almost matt on dorsal surface, males somewhat shiny), reduced on the most part of the clypeus and gula.

Head noticeably longer and narrower with respect to the pronotum, frons smooth, frontal furrows well-marked, divergent posteriorly, reaching the level of anterior supraorbital punctures; neck without constriction posteriorly; eyes fairly large, convex, moderately prominent, with diameter as long as the combined length of segment I-II of the antennae, temporae short, as long as or shorter than half of eye diameter; paraorbital sulci moderately deep, encircling eyes behind; clypeus trapezoidal, separated from frons by fine suture, with anterior margin slightly concave; labrum rectangular; antennae moderately long, pubescent from second fourth of segment IV, the apex of terminal segment not reaching basal margin of pronotum; mentum transverse, deeply emarginate, with large labial pits, median tooth slightly bifid at tip, epilobes narrow, slightly projecting beyond lobes; submentum with medial setae, without lateral ones (Fig. 28).

Pronotum wide, transverse, sub-trapezoid, widest about middle, with margins distinctly, narrowly bordered (the bordering reduced in the middle quarter of apical margin, and sometimes in the middle of basal margin, just between the internal basal impressions); sides somewhat more constricted apically than basally, with two pairs of setiferous punctures, lateral punctures situated at about end of apical third, posterolateral ones situated near hind angles, near to lateral margin and close to basal margin; apical margin moderately emarginate, narrower than basal margin, fore angles rounded, moderately projecting; basal margin nearly straight, slightly concave in the middle, hind angles almost rectangular, rounded at tip; basal impressions somewhat variable in extension and size, internal ones always present, linear, narrow and falcate, diverging toward base, impunctate, deeper and longer than the outer ones, outer impression present or reduced becoming evanescent, when present then mostly faint, foveolate, somewhat punctate; disc slightly convex, midline well-impressed, long, not reaching both anterior and posterior margins.

Elytra sub-elongate, moderately convex, widest at about the second third, fused at suture; shoulders well-marked, obtusely angulate; basal margin complete, reaching stria 1 inwards, forming a very minute denticle at humerus; discal striae moderately impressed, impunctate, parascutellar striae distinct, striae 1-8 joining basal margin; intervals slightly flat, smooth, interval 3 with three to four (rarely two or five) setiferous punctures adjoining stria 3, with last puncture in posterior third of elytron, rarely in about middle of interval 3 (see also Variability); scutellar setiferous puncture present; hind wings reduced to small scales.

Prosternum, mesosternum, middle of metasternum, proepipleura, epipleura of elytra, and abdominal sternites (excl. sides of sternites 1-3) smooth, impunctate, proepisterna and sides of sternites 1-3 slightly punctured, mesepisterna, metepisterna, and sides of metasternum more or less roughly punctured; intercoxal process of prothorax subquadrate, distinctly bordered at sides and backwards; metaepisterna short, sub-quadrate, moderately narrowed toward behind, its anterior border longer than internal and posterior ones, as long as external border.

Abdominal sternites IV-VI with transverse basal sulci complete (continuous) and well-impressed, abdominal sternum VI with posterior margin rimmed throughout, with one pair of foveate setigerous punctures medially in males and two pairs in females.

Legs slender, relatively long; protibia apically moderately but abruptly enlarged at internal margin in males; mesotibia and metatibia straight in both sexes, mesotibia with slight inner callus distally in males; tarsomeres 1-5 glabrous dorsally, segment 5 setose ventrally; segments 1-3 of male protarsi moderately expanded.

**Figures 19–22.**
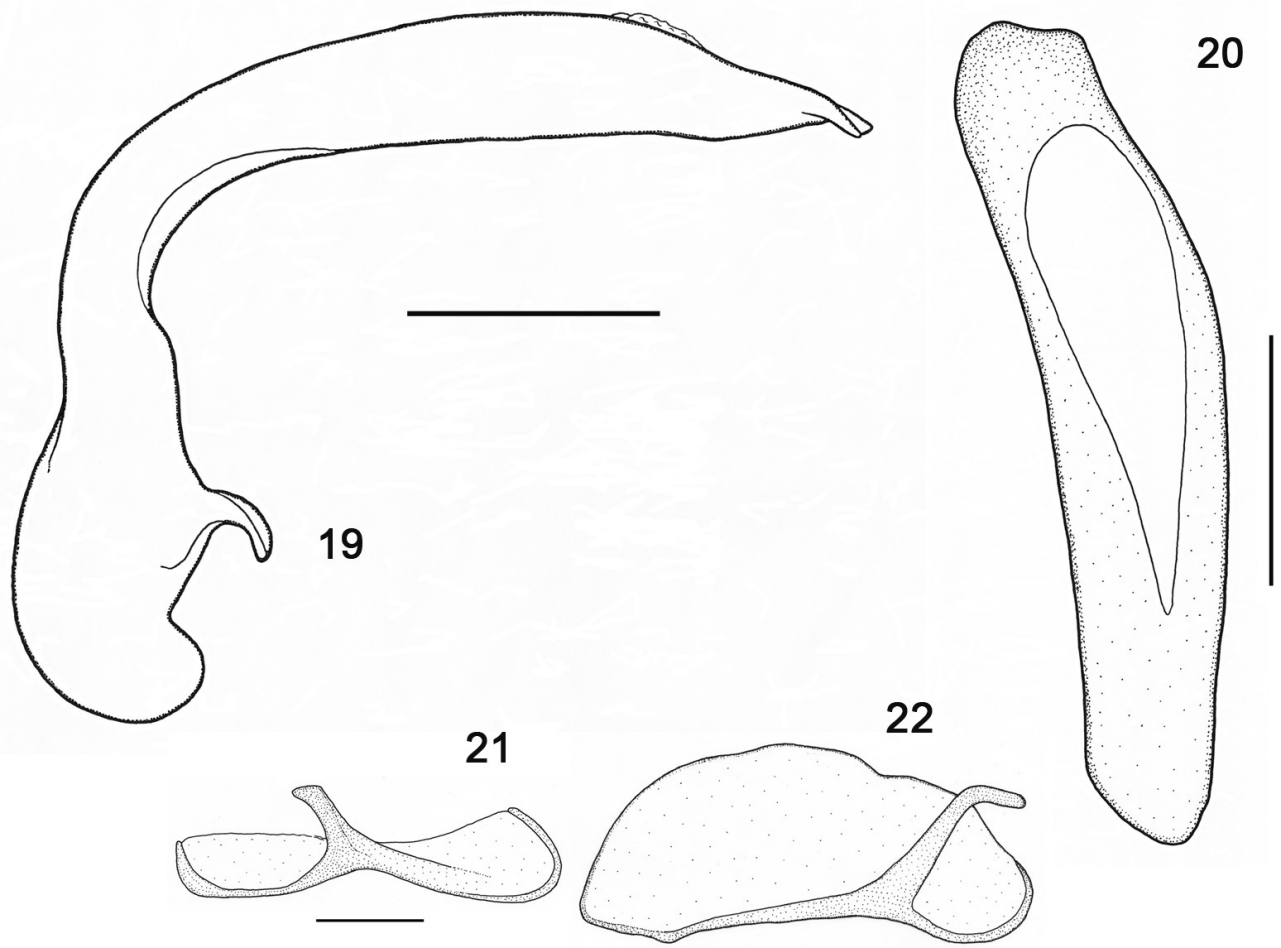
*Parorthomus socotranus* sp. n., paratype, male genitalia: **19** median lobe of aedeagus, left lateral aspect **20** median lobe of aedeagus, dorsal aspect **21** right paramere, internal face **22** left paramere, internal face. Scale bars = 0.5 mm (Figs 19–20), 0.2 mm (Figs 21–22).

Male genitalia (5 specimens dissected). Median lobe of aedeagus slender (Figs 19–20), narrower at middle, with basal part long, almost rectangularly bent behind apical part, narrowest at middle, from there toward apical lamella rectilinear, right external angle of apical lamella somewhat bent down, left external angle elevated (left lateral view), median lobe from middle part shifted to left, with right margin moderately convex, lengthwise appreciably elevated over left margin, left margin concave towards apex, apical lamella wide, rounded on left side, obtusely angled on right side, with a slight front concavity (dorsal view), ostium slightly deflected to right; right paramere narrow and elongate, smaller than left one, with a slanting lateral process (Fig. 21); left paramere conchoid (Fig. 22).

**Figures 23–24.**
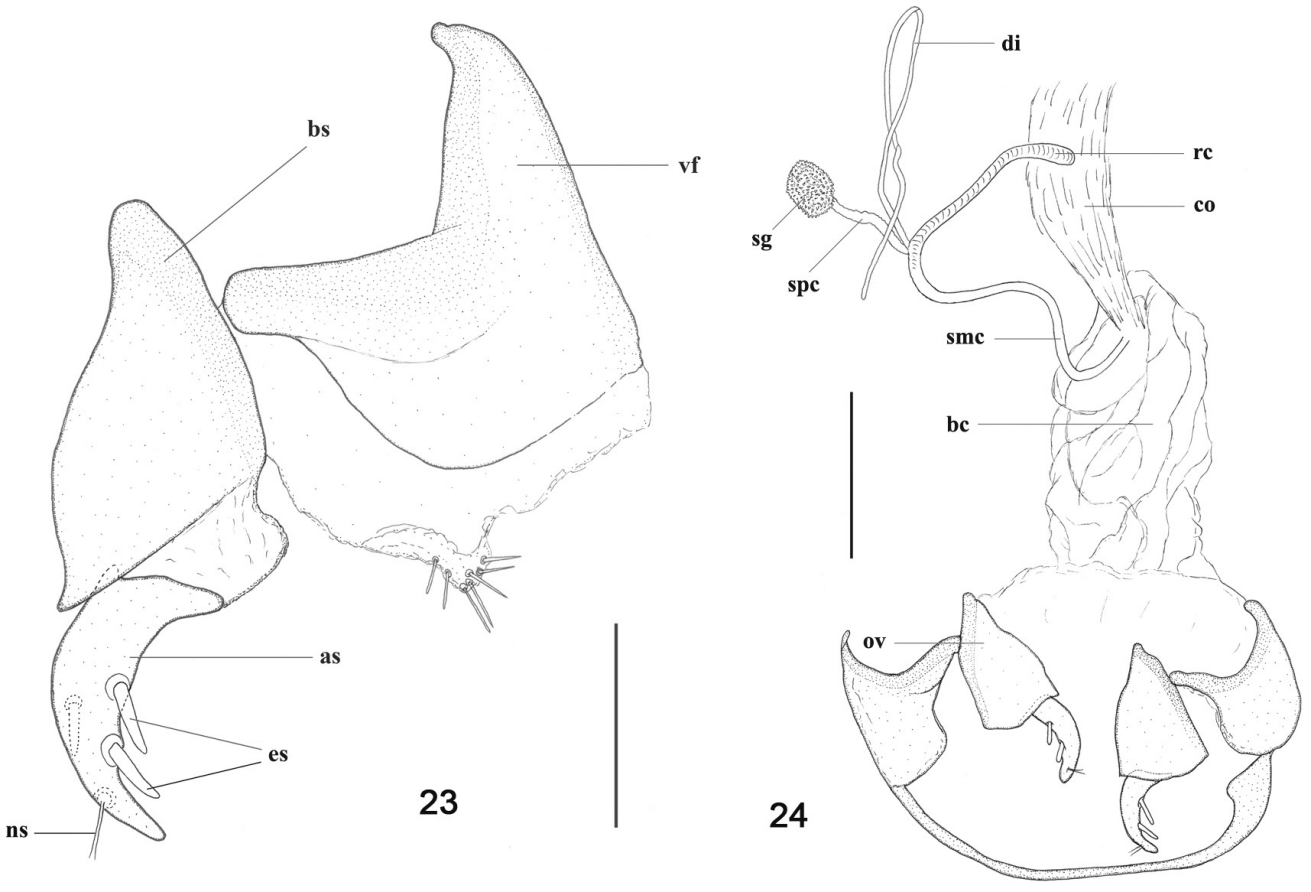
*Parorthomus socotranus* sp. n., paratype, female genitalia, ventral view: **23** left ovipositor **24** female reproductive tract (spermathecal complex and ovipositor). Legend: **as** apical stylomere; **bc** bursa copulatrix; **bs** basal stylomere; **co** common oviduct; **di** diverticulum; **es** ensiform setae; **ns** nematiform setae; **ov** ovipositor; **rc** receptaculum of spermatheca; **smc** seminal canal of spermatheca; **sg** appended gland of spermatheca; **spc** spermathecal canal; **vf** valvifer. Scale bars = 0.2 mm (Fig. 23), 0.5 mm (Fig. 24).

Female genitalia (3 specimens dissected). Ovipositor (Fig. 23), with valvifer more chitinized proximally and less distally, its distal margin having a setose and moderately chitinized area, basal stylomere large, conical, apical stylomere smaller, falcate, with two dorsolateral ensiform setae and one dorsomedial ensiform seta, sensorial pit distinct with two long nematiform setae; spermathecal complex (Fig. 24) with copulatory bursa proximally slightly gooseneck-like (this character not visible in Fig. 24), spermatheca with seminal canal and receptaculum slightly differentiated [undifferentiated type, according to Bousquet 1999: 35-36], receptaculum shorter than seminal canal, widened and slightly curved apically, appended spermathecal gland spherical, with elongate diverticulum.

##### Variability.

Interval 3 with three to four (rarely two or five) setiferous punctures adjoining stria 3, with last puncture in posterior third of elytron, rarely in about middle of interval 3. The number of punctures can increase to five or decrease to two, often the number of punctures of the left and the right elytron is unequal. Also the position of the punctures can somewhat vary. While the first two discal punctures always adjoin stria 3 (and so also the majority of the following punctures), sometimes the third puncture is located on the middle of interval or adjoins stria 2, rarely, the fourth discal pore is located on the middle of interval 3 or adjoins stria 2.

For variability of body size and indices see ‘Description’ and Table 1.

##### Etymology.

The specific epithet is an adjective, referring to Socotra, the island where the new species was collected.

##### Distribution.

Up to present only known from Socotra.

**Figures 29–31.**
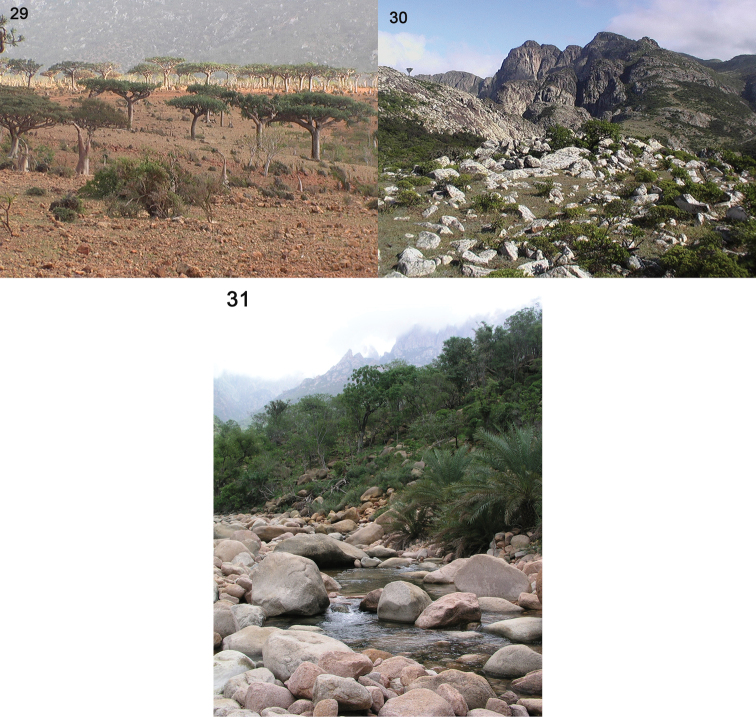
Habitats of *Parorthomus socotranus* sp. n. in Socotra. **29** Homhil protected area, November 2003 **30** Al Haghier Mts., December 2003 **31** Wadi Ayhaft, January 2004 (all photographs by JF).

##### Ecology.

A mesotopic to eurytopic epigeic beetle, collected from the end of September to the first ten days of February, and on higher ground (Hagher Mts., Scand Mt. env., 1450 m) some specimens were also found in June. From the list of localities, the species seems to be quite widespread across Socotra from the mouths of wadis (near or far off the water) till the highest mountains. Referring on Bezděk et al. (2012), the dominant habitat at most of the mentioned localities is high shrubland with dominant *Croton socotranus* and *Jatropha unicostata* (in higher altitude with intermixed *Boswellia* spp., *Dracaena cinnabari*, *Euphorbia arbuscula*, etc.) (Jiří Hájek, personal communication).

##### Systematic and biogeographic considerations.

The Socotra Archipelago is a Gondwanan continental fragment, which has experienced a long period of geological isolation. This landmass was separated from the Arabian plate during the rifting which began to open the Gulf of Aden in the Oligocene to Miocene epochs (d’Acremont et al. 2006). It is supposed that Socotra was isolated from Arabia at least 16 million years ago (d’Acremont et al. 2010). The high level of endemism found among the insects in Socotra (Batelka 2012) is in accordance with the estimated geological age and the supposedly continuous stability of its ecosystems.

At present, it is difficult to ascertain whether *Parorthomus socotranus* sp. n. derived from ancestral populations on the Arabian mainland that probably reached Socotra by transoceanic dispersal in relatively recent geological times, or it is a descendent of an ancestor and evolved *in situ* in the course and after the separation of the island. Notwithstanding, a few taxonomic and biogeographic facts are consistent with the hypothesis that the species is not a phyletically young descendant of continental populations.

Combinations of distinguishing features (see ‘Diagnosis’, Key to the genera of the “African Series” of Euchroina) clearly distinguish the new genus from the other related genera. However, some character states: 1/ mentum tooth bifid (Fig. 28); 2/ sides of pronotum straight or slightly convex posteriorly (Fig. 18); 3/ elytra with setiferous punctures in interval 3 (Fig. 18); 4/ intercoxal process of prothorax bordered; 5/ abdominal sternites V-VII with transverse sulci, complete and well-impressed; 6/ tarsomeres 1-5 of all legs glabrous dorsally; 7/ segment 5 of tarsomeres setose ventrally; 8/ aedeagus with sides nearly equally broadened in the distal half, with apical lamella wide, nearly rounded at tip (Fig. 20); 9/ appended spermathecal gland with diverticulum (Fig. 24), show that the new species may be related to two geographically “close” genera, the Afrotropical *Abacillodes* and the Mediterranean *Orthomus*. The two species from the first genus and the new species share characters 1-3 and 5-8. Some species from the second genus and *Parorthomus socotranus* sp. n. divide states 1-7 and 9 between, while the last taxon and *Orthomus velocissimus* s.l. possess all the listed character states.

**Figures 25–28.**
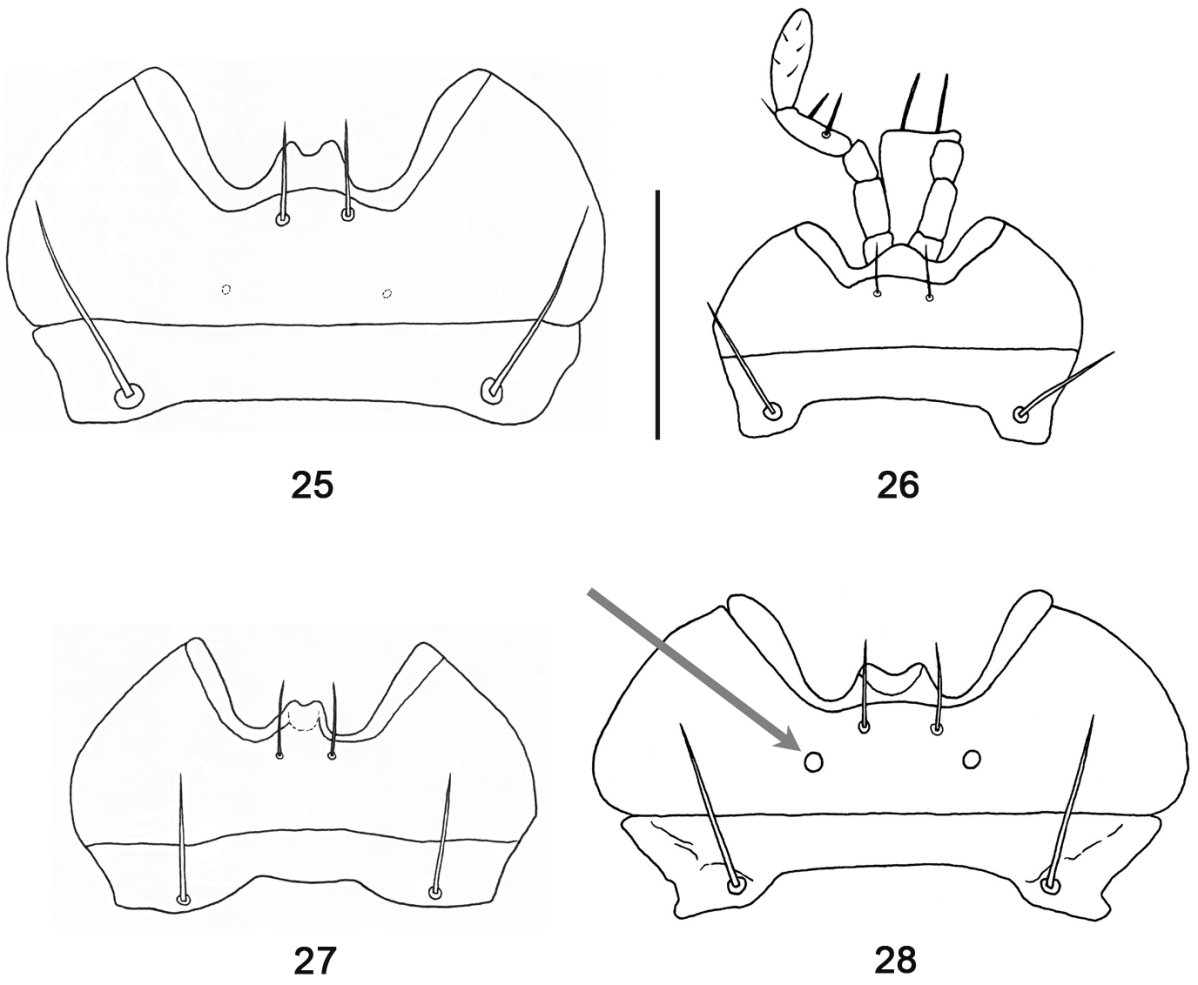
Mentum and submentum, ventral aspect (gray arrow indicating labial pits): **25** *Orthomus barbarus barbarus* (Dejean, 1828), female, Spain, “Laguna Salinas (Alicante)” **26** *Abacillius basilewskyi* Straneo, 1962, holotype **27** *Abacillodes jocquei* Straneo, 1988, holotype **28** *Parorthomus socotranus* sp. n., male paratype, “Fimihin”. Scale bar = 0.5 mm.

The median lobe with sides nearly equally broadened along the distal half and apical lamella wide, rounded or semi-rounded at tip in the new species looks alike the median lobe in the Afrotropical *Abacillodes* (see Straneo 1988: 482, Fig. 1d, 484, Fig. 2b), as well as those in some Mediterranean taxa of *Orthomus* (see Mateu 1957, laminas I-IV, Machado 1992: 262, Fig. 100, Wrase and Jeanne 2005: 894–896, Figs 1b, 2b, 3b, 4b, 5b, 6b, 7b, Pupier and Coulon 2013: 224, Figs 1c–e, 225, present paper Figs 5–8). In contrast, the median lobe of *Parorthomus socotranus* sp. n. has the distal half considerably curved to the left in dorsal aspect. Hitherto all the representatives of *Orthomus* and *Abacillodes* have the median lobe of the aedeagus straight or nearly straight. Based upon the condition in the other continental, African and Eurasian euchroines (aedeagus of *Abacillius* and *Trichopedius* not yet known or described), we consider the bent aedeagus to be an apotypic character in *Parorthomus* gen. n. The median lobes in the “*gracilipes*” group of *Nesorthomus*, with *Nesorthomus gracillipes* (Wollaston, 1854) and *Nesorthomus berrai* Battoni, 1987, have also the above discussed character state, more pronounced in the former and less pronounced in the second species (see Sciaky 1988: Figs 1b, 2b, Machado 1992: 264, Fig. 101A, Donabauer 2008: 111, Figs 1b, 2b, Serrano et al. 2009: 30, Figs 9b, 9d). This case is an instance of convergency. Change in this state has taken place independently in *Nesorthomus*, since the other six species from the genus have a straight median lobe of the aedeagus.

Besides, we infer that the presence of three to four discal setiferous punctures (by exception, two or five punctures on one elytron only) in the elytral interval 3 or stria 3, with the last puncture in posterior third of elytron, is another clear apotypic feature in the new taxon. This state occurs in no other species among the Old World Euchroina, except *Parorthomus socotranus* sp. n. The most species of the subtribe have two discal punctures in the elytral interval 3, as the second one lies at the posterior third of elytron. The species of *Abacillius* have no discal punctures on the elytron.

*Parorthomus socotranus* sp. n. has a unique combination of two apotypic characters, distal third of the aedeagus considerably curved to the left in dorsal aspect (i), and presence of 3-4 discal setiferous puncture in elytral stria 3/interval 3, with the last puncture situated in the posterior third of elytron (ii), which is indication for a long-time existing isolation and merit surely the erection of an own genus. The absence of close relative/s sharing together with the new species these two marked structural features exclude a close relationships and suggests that we deal with an ancient lineage which most probably arisen within the basic stem of the “African Series” of Euchroina (according to Will 2006) long time ago. As well, the lack of extant relatives, akin to the new species, in the Arabian Peninsula or somewhere else is a strong biogeographic argument, which certainly excludes geologically recent migration.

In spite of all, special character states and main ecologic preference in *Parorthomus socotranus* sp. n. suggest that it is phylogenetically closer to *Orthomus* and *Abacillodes* than to any other genus. The exact phylogenetic position within the euchroines can be disclosed only after investigation of more taxa, especially from the Old World, including also genetic technics and providing cladistic analysis, this could probably also identify its sister taxon.

The presence of large labial pits on the mentum is a trait in the new taxon that is worth noting. Each pit has a distinct, deep aperture, its diameter wider than the diameter of the labial pore, and both are situated more medially (Fig. 28). The distinct labial pits, destined to a particular function of use, seem to be a plesiotypic condition in Pterostichini (Bousquet 1999: 33), as well in the Nearctic euchroines (Frania and Ball 2007: 120). The species of *Abacillius*, *Abacillodes* and *Orthomus* possess no or small labial pits (Figs 25–27). In the second case, they have indistinct, shallow apertures, diameters similar to or smaller than the diameters of the labial pores, and both are situated more basally.

So far, 53 species of ground beetles have been recorded from the Socotra Archipelago (Felix et al. 2012, present work). *Parorthomus socotranus* sp. n. is the only representative of the tribe Pterostichini and the second carabid endemic form at genus level found in this insular fragment (Wranik 2003, Felix ibid.).

### Additionally examined material

#### Abacillius basilewskyi

Taxon classificationAnimaliaColeopteraCarabidae

Straneo, 1962

Fig. 26

Abacillius basilewskyi Straneo, 1962: 53 (type locality: “Natal, Drackensberg, Little Berg Summits, Themeda Grasslands, 5500–6000 ft., Cathedral Peak, Forestry reserve”)

##### Type material.

Holotype ♀, “Holotypus” [printed on salmon colored label], “Little berg summits Themeda Grassland 5500–6000 ft.” [printed], “cathedral peak forestry reserve. natal drakensberg. March 1959 B. R. & P. J. Stuckenberg” [printed], “Col. Mus. Congo don. B. Stuckenberg Coll. P. Basilewsky” [printed & handwritten], “*Abacillius Basilewskyi* n.sp. S.L. Straneo det. 1960 Holotypus“ [printed & handwritten], “RMCA ENT 000019508” [printed] (MRAC).

##### Remarks.

Straneo (1949: 7, 1958: 404) recorded that the onychium of the tarsi in *Abacillius aculeatus* is glabrous beneath. Subsequently, he described the same characteristics for *Abacillius basilewskyi* (Straneo, 1962: 54). However, the study of the holotype of the latter species revealed that it possesses the onychium finely setose beneath.

#### Abacillodes jocquei

Taxon classificationAnimaliaColeopteraCarabidae

Straneo, 1988

Fig. 27

Abacillodes jocquei Straneo, 1988: 483 (type locality: “Lichenya Plateau, 2000 m, Mount Mulanje”)

##### Type material.

Holotype ♂, “Holotypus” [printed on salmon colored label], “Lichenya Plateau 2000m 5/24.XI.1981” [printed], “Coll. Mus. Tervuren Malawi South. Reg. Mount Mulanje XI. 1981 - R. Jocqué” [printed], “Holotypus *Abacillodes jocquei* Str.” [printed & handwritten on red label], “*Abacillodes jocquei* n. sp. det. S.L. Straneo 1987 Holotypus ♂” [printed & handwritten], “RMCA ENT 000019509” [printed] (MRAC).

**Remarks.** A striking characteristic in the type species of *Abacillodes*, *Abacillodes jocquei*, are the elytral intervals 2, 4, and 6, significantly wider than the adjacent uneven intervals. But, this character state is not present in *Abacillodes malawianus*, thus it can not be used as a generic distinguishing mark.

#### Abacillodes malawianus

Taxon classificationAnimaliaColeopteraCarabidae

Straneo, 1988

Abacillodes malawianus Straneo, 1988: 483 (type locality: “Lichenya Plateau, 2000 m, Mount Mulanje”)

##### Type material.

Holotype ♀, “Holotypus” [printed on salmon colored label], “Lichenya Plateau 2000m 15/17.XI.1981” [printed], “Coll. Mus. Tervuren Malawi South. Reg. Mount Mulanje XI. 1981 - R. Jocqué” [printed], “Holotypus *Abacillodes malawianus* Str.” [printed & handwritten on red label], “*Abacillodes malawianus* n.sp. det. S.L. Straneo 1987 Holotypus ♂” [printed & handwritten], “RMCA ENT 000019510” [printed] (MRAC).

##### Remarks.

Straneo (1988: 483) stated the holotype is a male. Actually, it is a female specimen having protarsomeres not dilated and last abdominal sternite with four marginal setiferous punctures.

## Supplementary Material

XML Treatment for Orthomus berytensis

XML Treatment for Orthomus longior

XML Treatment for Orthomus longulus

XML Treatment for Orthomus velocissimus akbensis

XML Treatment for Parorthomus

XML Treatment for Parorthomus socotranus

XML Treatment for Abacillius basilewskyi

XML Treatment for Abacillodes jocquei

XML Treatment for Abacillodes malawianus
